# A delayed endothelial–glial mechanism for CGRP-induced migraine

**DOI:** 10.1186/s10194-026-02455-3

**Published:** 2026-07-16

**Authors:** Jochen K. Lennerz, Karl Messlinger

**Affiliations:** 1 Natera, San Carlos, CA USA; 2 Institute of Physiology and Pathophysiology, Friedrich-Alexander-Universität Erlangen-Nürnberg, Erlangen, Germany

**Keywords:** Migraine generation, CGRP receptors, Trigeminovascular system, Trigeminal ganglion neurons, Schwann cells, Nitric oxide, Reactive oxygen species, Transient receptor potential channels, ATP-sensitive potassium channels, Purinergic receptor

## Abstract

CGRP is a key mediator in migraine and an important component of trigeminovascular signaling in primary headache disorders. Yet how CGRP induces delayed migraine-like pain after systemic infusion remains unresolved. Despite strong clinical and experimental evidence for its role, the mechanisms linking CGRP signaling to trigeminovascular nociception are still unclear. Based on a comprehensive review of the experimental literature, we propose a hypothesis, in which CGRP initiates a delayed signaling cascade that ultimately sensitizes trigeminal afferents. In this framework, CGRP promotes not only arterial vasodilatation but activates glial responses involving transcription-dependent processes and ion channel modulation that evolve over hours and provide a mechanistic explanation for the delayed onset of migraine-like pain. We further propose that peripheral endothelial–glial signaling may be amplified at ganglionic and spinal levels, contributing to trigeminovascular sensitization. In spontaneous migraine attacks, CGRP release appears as a consequence of primary afferent excitation through an excess of nitric oxide species activating transient receptor potential channels, most likely initiated by an endothelial dysfunction through metabolic disturbances including cortical spreading depression. These peripheral mechanisms do not exclude central changes that govern the periodic susceptibility of migraine and cluster headache attacks. Our model integrates existing observations regarding CGRP biology, delayed migraine induction, and glial signaling into a unified framework. We reframe migraine generation as a neuro-glial process and highlight new targets for therapeutic intervention.

**Supplementary Information:**

The online version contains supplementary material available at 10.1186/s10194-026-02455-3.

## General characteristics of CGRP and neurogenic inflammation

Calcitonin gene-related peptide (CGRP) is a 37 amino acid neuropeptide with multiple functions [1, 2]. It exists in two isoforms; α-CGRP is derived from the CALC_A_ gene and is mainly expressed by small to middle-sized afferent neurons all over the body and in some central neurons, while β-CGRP is derived from the CALC_B_ gene and is mainly expressed by intrinsic neurons of the gut [3, 4]. The two isoforms differ in three amino acids, but their pharmacological properties are nearly identical [5]. In this review, the term CGRP refers to the alpha form.

CGRP producing afferents are mostly, although not exclusively, regarded as nociceptive and most of them belong to C-fiber afferents with unmyelinated fibers [6]. CGRP is released upon noxious stimulation or direct depolarization from the peripheral and central terminals of afferent neurons [7–10] and, at least experimentally, also from the cell bodies of dorsal root ganglion and trigeminal ganglion neurons by exocytosis, which is induced by an increase in cytoplasmic calcium [8, 11–14]. Thus, CGRP is widely distributed in nociceptive pathways and is readily released under conditions relevant for pain generation. In sensory endings, which lack significant calcium stores, this mechanism can be induced by calcium inflow through unspecific receptor cation channels, particularly the transient receptor potential channels vanilloid 1 (TRPV1) and ankyrin 1 (TRPA1), or voltage-gated calcium channels [14–16]. Thus, CGRP release can be initiated by local nociceptor stimulation and does not require action potential generation.

Content and release of CGRP can be determined and quantified with enzyme-linked immunoassays or radioimmune assays in extracellular fluids and in nearly all tissues innervated by CGRP releasing afferents [17–21]. However, interpretation of reported CGRP levels requires caution, as assay performance and differentiation between α- and β-CGRP may vary between studies.

CGRP is co-localized in large part with other neuropeptides like substance P and neurokinin A in nociceptive primary afferents [22–24]. The collective release of these neuropeptides upon noxious stimulation causes the so-called neurogenic inflammation, which consists of vasodilatation and increased blood flow caused by CGRP, protein plasma extravasation caused by substance P and neurokinin A, and other effects like degranulation of mast cells [25, 26, 29–32]. These responses become noticeable as flare reaction (reddening), wheal (swelling) and itch or pain in different experiments [27, 28].

Injection of CGRP into human skin causes flare but no other symptoms [29, 30]. Likewise, local neurogenic inflammation in human skin, e.g. induced by electric stimulation, causes flare but not a wheal, indicating that under these conditions substance P is either not sufficiently released or does not cause significant plasma extravasation [31, 32]. Importantly, local administration of CGRP induces vascular responses but does not cause pain, highlighting a central paradox that motivates the present hypothesis: despite its established role in migraine, CGRP alone does not appear to directly generate pain at sites of local release.

## CGRP, trigeminal nociception and headaches

CGRP is long known as a key mediator in the generation of migraine pain and trigeminal autonomic cephalalgias (TACs) [33]. CGRP has been found at elevated levels in the venous outflow from the head during migraine and cluster headache attacks [34] as well as in saliva [35] and tear fluid [20]. However, interpretation of CGRP measurements requires consideration of methodological variability and assay-specific limitations, including differences in isoform detection. Infusion of CGRP can induce delayed migraine-like headaches in migraine patients [36, 37] and delayed cluster attacks in cluster headache patients [38]. Interestingly, in cluster headache, CGRP appears capable of triggering attacks primarily during active cluster periods, suggesting that additional central modulatory mechanisms may influence susceptibility. These clinical observations strongly support a causal role of CGRP in migraine generation, which is the subject of several excellent recent reviews [39, 40].

Several therapeutic options further confirm the importance of CGRP signaling, including the use of triptans, gepants, and monoclonal antibodies targeting CGRP release, CGRP or its receptors, and all are effective in migraine therapy [41–43, 59]. Also, many preclinical experiments have confirmed CGRP release from trigeminal afferents and the vascular effects of CGRP [9, 10, 44–51, 52–58].

Despite extensive evidence, several paradoxical observations remain unresolved. Application of CGRP to the meninges of female rodents has been reported to induce periorbital mechanical allodynia, a migraine-related symptom frequently examined in pre-clinical experiments [52]. However, intravenous or local administration of CGRP in humans is not acutely painful [22] and does not cause activation and sensitization of meningeal nociceptors without further intervention in animals [53]. Although the immediate effects of intravenous CGRP infusion are predominantly vascular, including flushing, palpitations, warm sensation, increased heart rate, and reduced mean arterial pressure due to arterial vasodilatation [54], CGRP also induces migraine-relevant phenotypes such as migraine-like pain and photophobia, indicating biological actions beyond vasodilatation alone. To reconcile these observations, we focus on four unresolved questions that collectively motivate the mechanistic framework proposed in this review:

- Question 1: Why is the infusion of CGRP in migraineurs followed by migraine-like pain?
- Question 2: Why does CGRP induce migraine-like pain but no other pains in the body?
- Question 3: Why is the migraine-like headache induced by CGRP delayed?
- Question 4: Which initial mechanisms activating trigeminovascular nociceptors induce migraine attacks?

These four questions form the central rationale for the present review. We propose that they can be reconciled within a unified endothelial–glial model of CGRP signaling. Table 1 and Supplementary Fig. 1 summarize the proposed answers, the underlying biological mechanisms, and the manuscript sections in which the supporting evidence is discussed.

**Table 1:** Conceptual framework. A unified mechanistic framework to four unresolved questions in CGRP-induced migraine

Question	Proposed answer	Key mechanism	Relevant sections
Question 1**.** Why does infusion of CGRP in migraineurs cause migraine-like pain?	CGRP initiates a delayed endothelial–glial signaling cascade that ultimately sensitizes trigeminovascular afferents.	CGRP → endothelial signaling → NO/HNO → TRPA1 activation → glial responses → delayed afferent sensitization.	*3. General characteristics of CGRP receptors and intracellular signaling; 6. Mechanisms releasing CGRP in trigeminal nociception and headache; 9. Role of Schwann cells in NO-TRPA1-CGRP signaling; 11. Possible role of NO-TRPA1-CGRP signaling in the trigeminal ganglion; Conclusions.*
Question 2. Why does CGRP induce headache but not other pains in the body?	The trigeminovascular system is uniquely enriched for CGRP-containing perivascular afferents and associated signaling pathways.	High density of CGRP-positive afferents surrounding intracranial arteries provides a specialized substrate for headache generation.	*4. CGRP releasing nerve fibers and their function in the trigeminovascular system; 5. Presence of CGRP receptors in the trigeminovascular system; Conclusions.*
Question 3. Why is CGRP-induced migraine delayed?	CGRP signaling involves transcription-dependent glial responses that evolve over hours rather than minutes.	cAMP/PKA/ERK signaling induces delayed cellular responses, including expression of pro-nociceptive mediators.	*3. General characteristics of CGRP receptors and intracellular signaling; 8. Neuronal signaling of CGRP in the trigeminovascular system; 9. Role of Schwann cells in NO-TRPA1-CGRP signaling; 11. Possible role of NO-TRPA1-CGRP signaling in the trigeminal ganglion; Conclusions.*
Question 4. Which mechanisms initially activate trigeminovascular nociceptors during migraine?	Multiple vascularly derived mediators may initiate trigeminovascular signaling, including NO/HNO-TRPA1 signaling, ATP-mediated purinergic signaling, and cortical spreading depression-associated vascular responses.	Endothelial dysfunction, oxidative stress, ATP release, NO/HNO signaling, and cortical spreading depression may converge on activation of trigeminal afferents.	*6. Mechanisms releasing CGRP in trigeminal nociception and headache; 7. Possible role for ATP in CGRP release; 10. Role of cortical spreading depression in CGRP signaling; Conclusions.*

To facilitate future experimental validation, Table 2 summarizes the principal testable predictions generated by the proposed model together with the current preclinical and clinical evidence supporting each prediction.

**Table 2:** Experimental roadmap. Principal testable predictions arising from the proposed endothelial–glial model of CGRP-induced migraine

Mechanistic prediction	Supporting preclinical evidence	Clinical observations / opportunities for validation	Relevant section(s)
CGRP induces delayed migraine through transcription-dependent signaling rather than direct nociceptor activation.	CGRP activates cAMP/PKA/ERK/CREB signaling, induces gene expression in Schwann cells and neurons, but does not directly activate or sensitize meningeal afferents.	Intravenous CGRP reliably induces delayed migraine-like headache in susceptible individuals. **Prediction**: interventions that interrupt downstream transcriptional responses should attenuate delayed migraine despite preserved vasodilatation.	*3. General characteristics of CGRP receptors and intracellular signaling*
The trigeminovascular system is uniquely susceptible because of its enrichment in CGRP-containing afferents and receptors.	High density of CGRP-positive afferents and receptor expression around intracranial arteries compared with most peripheral tissues	Explains why systemic CGRP preferentially induces headache rather than pain elsewhere. **Prediction**: regional differences in receptor distribution should correlate with susceptibility to migraine generation.	*4–5. Trigeminovascular anatomy and receptor distribution*
Endothelial NO/HNO signaling represents the initiating event of the proposed cascade.	NO donors produce vasodilatation and delayed trigeminal activation; HNO is a potent TRPA1 agonist and promotes CGRP release more effectively than NO. Endothelial NOS provides a plausible endogenous source of NO.	NO donors reproducibly provoke delayed migraine-like headache. **Prediction**: selective interruption of endothelial NO/HNO–TRPA1 signaling should prevent migraine initiation while preserving downstream pathways.	*6. Mechanisms releasing CGRP in trigeminal nociception and headache*
ATP contributes to downstream activation of trigeminovascular afferents following vascular signaling.	Extracellular ATP activates trigeminal afferents through purinergic receptors and can be released from vascular and glial cells.	Direct ATP provocation studies are currently lacking. **Prediction**: inhibition of purinergic signaling should reduce afferent activation downstream of vascular stimulation.	*7. Possible role for ATP in CGRP release*
Neuronal CGRP signaling contributes primarily to delayed sensitization rather than acute nociceptor activation.	CGRP activates intracellular signaling pathways associated with transcriptional regulation and neuronal plasticity without directly exciting nociceptors.	Delayed rather than immediate headache after CGRP infusion supports a secondary signaling mechanism. **Prediction**: delayed neuronal transcriptional responses should parallel migraine onset.	*8. Neuronal signaling of CGRP in the trigeminovascular system*
Schwann cells amplify endothelial signaling and contribute to delayed trigeminovascular sensitization.	Schwann cells express CGRP receptors, activate ERK-dependent signaling, and induce inflammatory mediators including NOS.	No direct clinical validation currently available. **Prediction**: selective modulation of Schwann-cell signaling should reduce delayed sensitization without preventing the initial vascular response.	*9. Role of Schwann cells in NO–TRPA1–CGRP signaling*
Cortical spreading depression, oxidative stress, and endothelial dysfunction provide upstream initiating events for the cascade.	CSD, reactive oxygen species, H₂S, and TRPA1 activation converge on endothelial and trigeminal signaling pathways capable of promoting CGRP release.	Indirect clinical evidence links endothelial dysfunction and CSD with migraine susceptibility. **Prediction**: biomarkers of oxidative or endothelial stress should correlate with activation of the proposed cascade.	*10. Role of cortical spreading depression in CGRP signaling*
Ganglionic and spinal glial mechanisms amplify and sustain trigeminovascular sensitization.	Satellite glial cells and Schwann cells express CGRP receptor components and participate in neuron–glia communication within the trigeminal ganglion and spinal trigeminal system.	Direct clinical validation remains limited. **Prediction**: interventions targeting ganglionic glial signaling should attenuate sustained migraine pain rather than the initiating event.	*11. Possible role of NO–TRPA1–CGRP signaling in the trigeminal ganglion*
Therapeutic interruption of the cascade should identify the most critical mechanistic nodes.	CGRP antagonists reduce trigeminovascular activation in experimental models, whereas non-selective NOS inhibition attenuates migraine-related signaling	Clinical efficacy of gepants and anti-CGRP monoclonal antibodies validates downstream blockade; the more limited success of NOS-directed therapies helps refine the proposed hierarchy of the cascade. Future comparison of interventions targeting endothelial, neuronal, and glial components may further define the critical initiating and amplifying mechanisms.	*Conclusions*

The table summarizes the major mechanistic predictions generated by the proposed model and links each prediction to the available preclinical evidence and corresponding clinical observations or opportunities for future experimental validation. The varying degree of supporting evidence illustrates which components of the proposed signaling cascade are well established and which remain hypothetical

The framework outlined in Tables 1 and 2 serves as a roadmap for the remainder of this review. The following sections examine the experimental and clinical evidence supporting each component of the proposed endothelial–glial cascade, progressing from receptor biology and vascular signaling to neuronal, glial, and ganglionic mechanisms before returning to an integrated conclusion.

## General characteristics of CGRP receptors and intracellular signaling

CGRP receptors are composed of three independently expressed proteins, the seven membrane-spanning calcitonin receptor-like receptor (CLR), the one membrane-spanning receptor activity-modifying protein 1 (RAMP1) and an intracellular component, the receptor component protein (RCP), which regulates CGRP signaling [55, 56]. In addition, the amylin-1 receptor can bind CGRP with similar affinity and may contribute to CGRP-mediated signaling [58]. Detailed discussion of receptor subtypes has been reviewed elsewhere [5, 59].

The intracellular metabolic cascades linked to all these receptors are largely identical. Binding of CGRP to its receptors induces adenylyl cyclase (AC), which increases cyclic adenosine monophosphate (cAMP) levels [4]. Increase in cAMP levels activates protein kinase (PKA), which can phosphorylate and activate multiple proteins such as ion channels and proteins with enzymatic activity [2]. In vascular smooth muscle, this pathway contributes to vasodilation through activation of potassium channels and membrane hyperpolarization [60–62].

Finally, CGRP can activate cAMP-dependent intracellular cascades to modify gene expression. In Schwann cells, CGRP has been observed to phosphorylate extracellularly regulated kinase (ERK) inducing gene transcription of inflammatory mediators [63]. In human keratinocytes, CGRP regulated the expression of vascular endothelial growth factor (VEGF) via ERK1/2 [64]. In trigeminal ganglion neurons, CGRP stimulated the phosphorylation of cAMP response element-binding protein (CREB), of stress-activated protein kinase p38 and of ERK, which has been shown to be blocked by the CGRP receptor antagonist olcegepant [65].

Importantly, these transcription-dependent responses evolve over hours rather than minutes and therefore provide a potential mechanistic framework for Question 3, namely the delayed onset of migraine-like pain following CGRP exposure.

## CGRP releasing nerve fibers and their function in the trigeminovascular system

CGRP and its receptors seem to be especially important in the trigeminovascular system, which is regarded as the morpho-functional basis of headache and migraine generation [19, 66]. The term “trigeminovascular” characterizes the close functional relationship between blood vessels, particularly arterial vessels, and perivascular nerve fibers of trigeminal origin; this term is mostly, if not explicitly, referred to intracranial blood vessels, i.e. meningeal and cortical arterial vessels; although extracranial structures are also innervated by trigeminal afferents and may contribute to headache generation [67–69]. The trigeminovascular innervation consists mainly of unmyelinated C-fibers, while the minority of fibers (10–20%) are thinly myelinated Aδ-fibers [70–73]. The central extensions of the trigeminal afferents form the trigeminal nerve, which enter the contralateral brainstem at the pontine level [74]. C- and Aδ-fibers descend to the medulla and synapse on second-order neurons within the spinal trigeminal nucleus and upper cervical spinal cord [75–79].

**Fig. 1:**
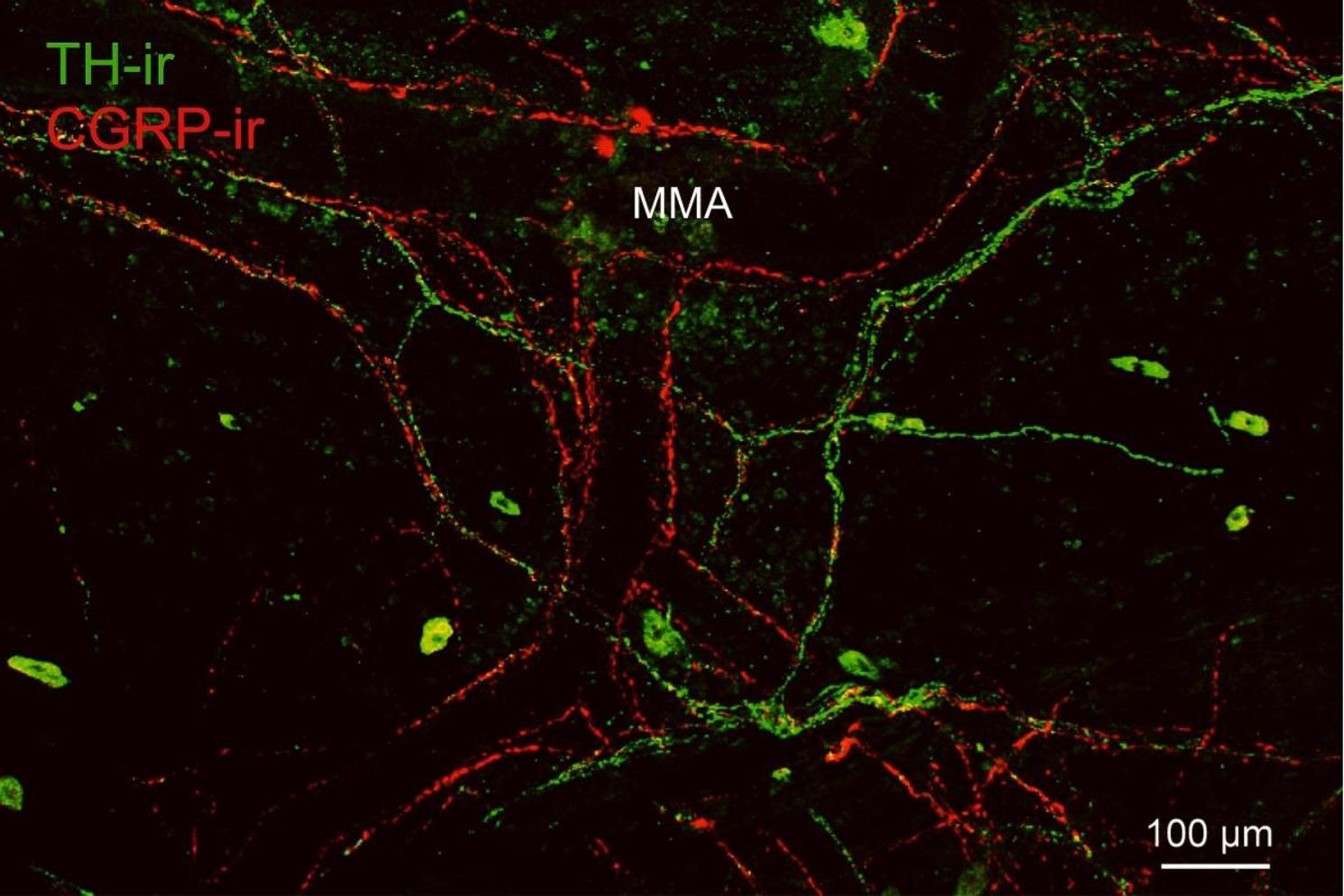
Bundles of CGRP immunoreactive (CGRP-ir) and tyrosine hydroxylase immunoreactive (TH-ir) nerve fibers accompany the branched middle meningeal artery (MMA) in the rat parietal dura mater; TH-ir cells represent mast cells. Courtesy of S. Uchida and W. Neuhuber

A major proportion of intracranial afferents is peptidergic, mainly containing CGRP; retrograde tracing combined with immunohistochemistry in rat revealed that 32% of trigeminal afferents innervating cerebral arteries [80] and even 66% innervating the dura mater are CGRP-immunopositive [81]. These proportions are substantially higher than those reported in most peripheral tissues, suggesting a relative enrichment of CGRP-containing afferents within the intracranial trigeminovascular system (Fig. 1). This enrichment may provide a partial explanation for Question 2, namely why CGRP preferentially induces headache rather than pain in other tissues.

Single CGRP immunoreactive nerve fibers in the dura mater are closely associated with precapillary and arterial vessels, whereas capillaries appear largely devoid of direct innervation [71, 82, 83]. CGRP-immunopositive nerve fibers form club-like terminals at arterial vessels but also extend into the surrounding connective tissue [71].

The close association of CGRP releasing nerve fibers with arterial blood vessels indicates an important vascular function of CGRP, particularly in the cranial vasculature [54]. Indeed, CGRP is regarded as the most potent vasodilatory substance of intracranial arteries, followed by substance P and neurokinin A [84]. Activation of smooth vascular CGRP receptors increases intracellular cAMP and downstream signaling through protein kinase A, resulting in potassium-channel activation, smooth-muscle relaxation, and vasodilatation [85–87].

In addition to trigeminal afferents, intracranial arterial vessels are densely innervated by sympathetic postganglionic neurons (see Fig. 1) arising from the superior cervical ganglion and parasympathetic postganglionic neurons mostly arising from the sphenopalatine and otic ganglia [82, 88–91]. Sympathetic fibers releasing norepinephrine and neuropeptide Y (NPY) primarily promote vasoconstriction, whereas trigeminal and parasympathetic fibers release vasoactive mediators including vasoactive intestinal polypeptide (VIP), and pituitary adenylate cyclase-activating polypeptide (PACAP), which contribute to vasodilatation [92–95].

Taken together, the enrichment of CGRP-containing afferents around intracranial arteries and their intimate association with the trigeminovascular system suggest a unique anatomical substrate, through which CGRP signaling may preferentially generate headache, thereby providing a potential explanation for Question 2.

## Presence of CGRP receptors in the trigeminovascular system

The expression and distribution of CGRP receptor components have been demonstrated throughout the peripheral trigeminovascular system, including the dura mater, trigeminal ganglion, and spinal trigeminal nucleus [72, 96–99].

**Fig. 2:**
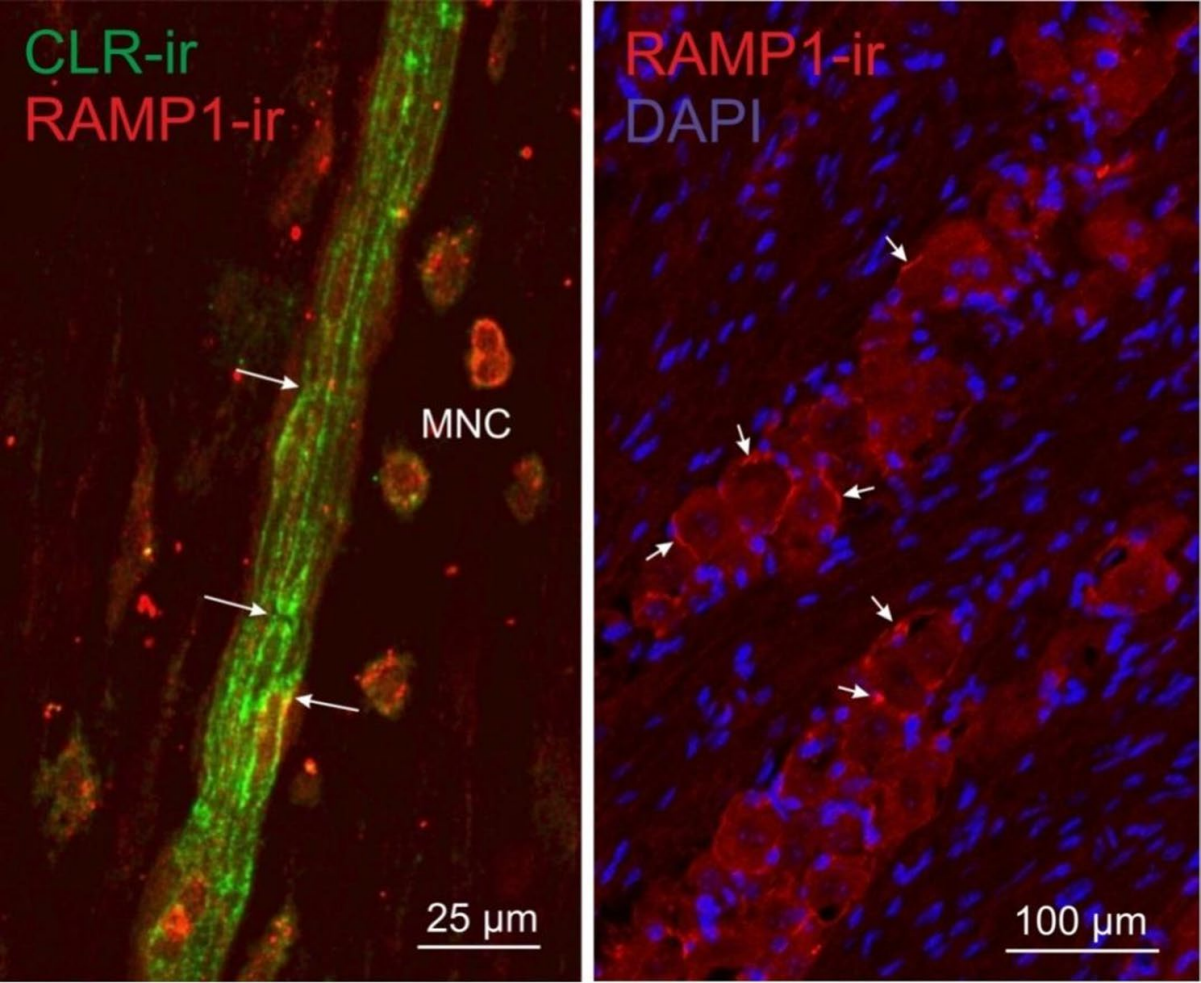
Left: Small nerve fibre bundle in the rat dura mater immunohistochemically processed for calcitonin receptor-like receptor (CLR-ir) and receptor activity-modifying protein 1 (RAMP1-ir). Overlay of both signals results in a yellowish color. The receptor components are seen in Schwann cells and are especially dense at the nodes of Ranvier (arrows). MNC, mononuclear cells. Courtesy of J. Lennerz and W. Neuhuber. Right: Rat trigeminal ganglion immunohistochemically processed for receptor activity-modifying protein1 (RAMP1-ir) and stained with DAPI for nuclear proteins. RAMP1-ir appears especially dense in structures around neurons, likely in satellite glial cells. Courtesy of M. Dux and B. Vogler

According to our studies in the rat [96] CGRP receptor components, particularly CLR and RAMP1, are widely distributed throughout the trigeminovascular system. In the dura mater, immunoreactivity for CLR and RAMP1 has been detected in Schwann cells of putative myelinated afferent fibers (Fig. 2, left). In the trigeminal ganglion, immunoreactivity for CGRP receptor components has been located to satellite glial cells (Fig. 2, right), Schwann cells and subsets of neurons, while colocalization with CGRP-containing neurons was extremely rare.

In the spinal trigeminal nucleus, CGRP receptor components were localized mainly to fibrous and glomerular structures, consistent with neuronal processes and primary afferent endings, while staining was largely absent from neuronal cell bodies and central glial elements. This distribution indicates that CGRP signaling in the brainstem is likely to occur predominantly at presynaptic sites, modulating nociceptive transmission [100, 101]. Together, these findings support the concept that CGRP receptors are positioned at multiple levels of the trigeminovascular system, including glial and neuronal compartments, providing several potential targets for CGRP-mediated modulation of trigeminal nociception.

Subsequent studies using human tissue and improved immunohistochemical and transcriptomic approaches have largely confirmed this distribution. In human trigeminal ganglia, CGRP receptor components, including CLR and RAMP1, have been detected predominantly in satellite glial cells and Schwann cells while also being present in substantial subset of neurons [97]. Reported neuronal expression levels vary across studies and methodologies, but available data support receptor localization in both neuronal and non-neuronal cellular compartments. This pattern supports a prominent role of neuron–glia signaling in CGRP-mediated effects. In addition, CGRP receptor expression has long been demonstrated in meningeal vascular smooth muscle and endothelial cells, consistent with vascular actions of CGRP [102, 103].

Recent single-cell transcriptomic and epigenomic analyses of human and mouse trigeminal ganglia have identified diverse neuronal, glial, immune, and vascular-associated cell populations, supporting the concept that CGRP signaling occurs within a complex multicellular network rather than a purely neuronal pathway [104, 105]. Taken together, these findings support the hypothesis that many CGRP-mediated effects may be amplified or propagated through glial and vascular intermediates, while not excluding direct actions on subsets of trigeminal neurons.

Overall, the available anatomical and molecular evidence supports a distributed CGRP receptor network across peripheral, ganglionic, and central components of the trigeminovascular system, providing multiple potential sites through which CGRP may influence migraine-related nociceptive signaling.

## Mechanisms releasing CGRP in trigeminal nociception and generation of migraine pain

**Fig. 3:**
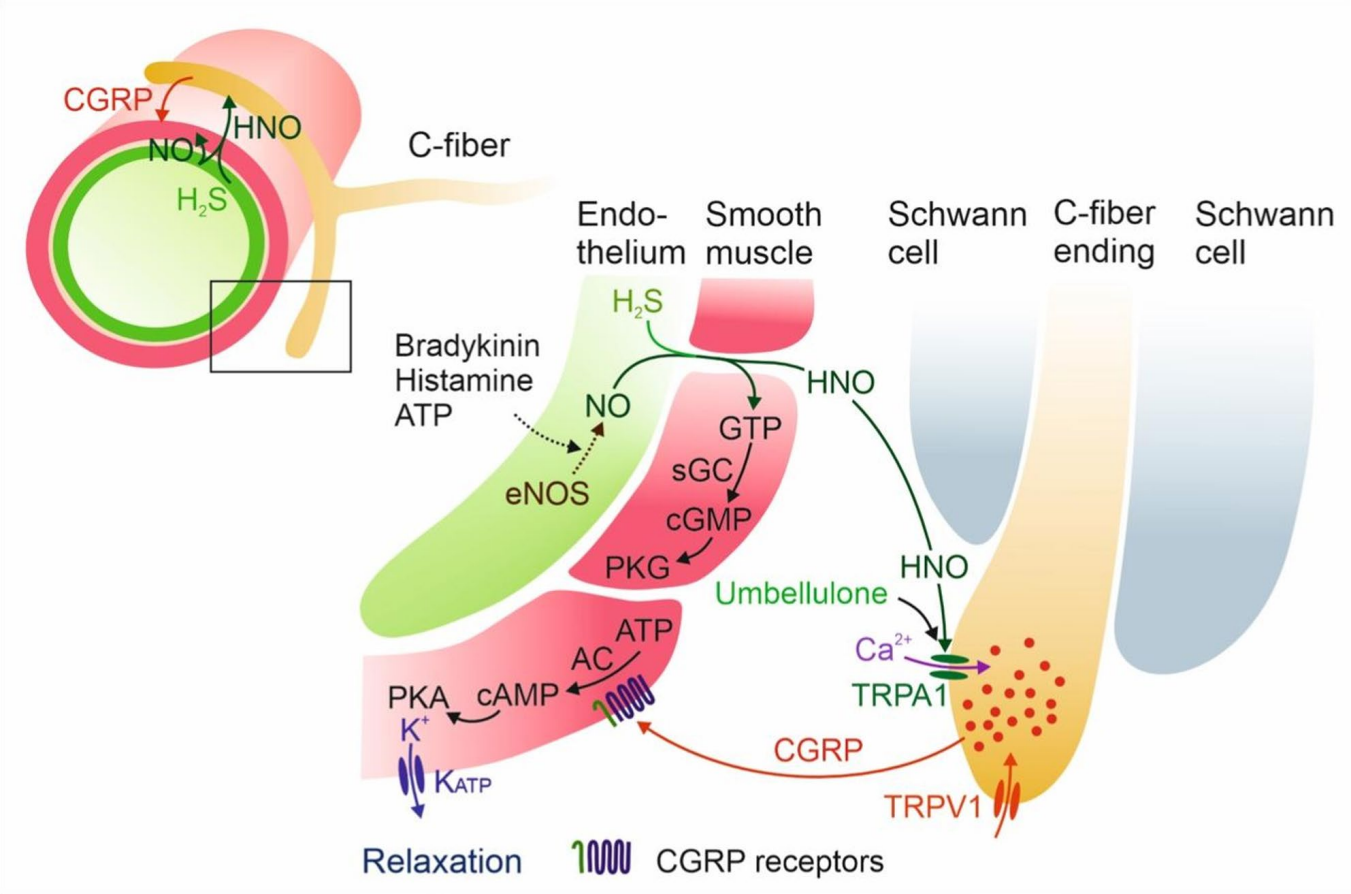
NO- and CGRP-dependent relaxation of vascular smooth muscle cells and NO-HNO-TRPA1 signaling to release CGRP from peptidergic afferents. Umbellulone activates TRPA1 receptors directly. AC, adenylyl cyclase; ATP adenosine triphosphate; cAMP/cGMP, cyclic adenosine/guanosine monophosphate; eNOS, endothelial NO synthase; GTP, guanosine triphosphate; H_2_S, hydrogen sulfide; HNO, nitroxyl (NO-); K_ATP_, ATP-sensitive potassium channel; NO, nitric oxide; PKA/PKG, protein kinase A/G; sGC, soluble guanylate cyclase; TRPA1/TRPV1, transient receptor potential ankyrin-1/vanilloid-1 receptor channels

CGRP release requires activation of peptidergic afferents and calcium influx, typically through TRPV1 or TRPA1 channels. One clue may come from umbellulone, a headache-inducing ketone found in the Californian “headache tree” (*Umbellularia californica*). Umbellulone has been shown to activate TRPA1 expressing HEK293 cells and rat trigeminal ganglion neurons and to release CGRP from trigeminal ganglion and dura mater [106] (Fig. 3).

TRPA1 can also be activated by endogenous mediators, including unsaturated fatty acids and nitric oxide (NO)-related species, particularly nitroxyl (HNO), which can be generated from NO and hydrogen sulfide (H_2_S) [107, 108]. HNO is a potent TRPA1 agonist. The HNO donor Angeli’s salt induces calcium influx and robust CGRP release, whereas the NO donor DEA NONOate is comparatively ineffective [108].

Under natural conditions, NO is generated in vascular endothelial cells through endothelial NO synthase (eNOS), which is activated by shear stress and inflammatory mediators such as bradykinin, histamine, and extracellular ATP [109] (Fig. 3). NO promotes arterial vasodilatation through activation of the sGC–cGMP–PKG pathway (Fig. 3). HNO may also be generated in dural arteries. NO and H_2_S, the precursors for HNO synthesis, have been shown by histochemical methods to be present in the endothelium of the rat middle meningeal artery [110]. We therefore hypothesize that HNO diffuses to adjacent perivascular afferents, activates TRPA1 channels, and promotes CGRP release. Released CGRP may then further enhance vasodilatation through vascular CGRP receptors, creating a positive-feedback loop within the trigeminovascular system (Fig. 3).

With regard to episodic and chronic migraine, we propose that NO-HNO-TRPA1-CGRP signaling may contribute to migraine generation and possibly trigger migraine attacks. An endothelial dysfunction of cerebral arterial vessels has been made responsible for an increased susceptibility for migraine [111–113], while an impaired vasodilatation through the imbalance of vasoconstricting (e.g. endothelin) and vasodilating mediators (e.g. NO) in arterial vessel walls has been observed [114, 115]. In addition, associations of polymorphisms for endothelial NO synthase with migraine manifestation have been reported [116–118]. We speculate that altered endothelial NO signaling may favor HNO-dependent activation of trigeminovascular afferents, particularly in tissues enriched for perivascular CGRP-containing fibers [88]. Consistent with a vascular contribution, migraine is also associated with an increased risk of several vascular disorders, including stroke [119].

Our proposed mechanism primarily focuses on extravascular actions of CGRP within the trigeminovascular system, where locally released CGRP can interact with vascular, glial, and neuronal targets. At the same time, the ability of intravenously administered CGRP to trigger migraine demonstrates that luminal exposure can initiate biologically relevant signaling cascades [37, 120]. Notably, the CGRP concentrations achieved during infusion studies are substantially higher than those measured in jugular venous plasma during spontaneous migraine attacks [34, 121, 122]. We therefore propose that a fraction of intravenously administered CGRP gains access to perivascular and ganglionic compartments, where it can interact with trigeminal targets. Conversely, CGRP measured in the venous circulation during migraine likely represents peptide that has already diffused away from its local sites of action. Thus, concentrations at perivascular release sites can be expected to be substantially higher than those detected in peripheral blood. This interpretation does not exclude a physiological role for circulating CGRP, particularly in mediating vasodilatation, which occurs at low picomolar concentrations [84]. These vascular effects are mediated by CGRP receptors on vascular smooth muscle and potentially also on endothelial cells, where CGRP signaling may stimulate endothelial NO synthase activity and NO production [123] (see Fig. 3).

Beyond blood vessels, functional CGRP receptors have recently been identified on endothelial cells of meningeal lymphatic vessels [124], extending the potential anatomical sites of CGRP action [125–127]. Notably, mice lacking these endothelial CGRP receptors showed reduced sensitivity to nitroglycerin, suggesting that lymphatic endothelial signaling may also contribute to migraine-relevant CGRP responses. While NO/HNO-dependent signaling provides one plausible mechanism linking vascular function to CGRP release, other endothelial mediators may also contribute to trigeminovascular activation.

## Possible role for ATP signaling in CGRP release

Extracellular ATP has also been proposed as an initiator of trigeminovascular signaling [128]. Shear stress on arterial endothelium has been shown to release ATP, mediated through stretch-activated cation channels of the PIEZO1 type [129]. ATP activates endothelial purinergic P2Y receptors, which may lead to the formation of NO and arterial vasodilatation [130, 131]. ATP may subsequently diffuse into the perivascular space and activate P2X receptors of sensory afferents, promoting CGRP release [132, 133]. Furthermore, there is experimental evidence that CGRP causes delayed sensitization, upregulation and gene expression of P2X receptors in trigeminal afferents [134–136]. ATP-sensitive mast cells may further amplify this response through release of histamine, ATP, and mast-cell tryptase [137–139]. Interestingly, H_2_S inhibits ATP-induced activation of trigeminal afferents despite causing mild direct activation itself [140].

## Neuronal signaling of CGRP in the trigeminovascular system

How CGRP can signal between afferent fibers in the trigeminovascular system remains incompletely understood. In a recent study, intra-carotid infusion of CGRP into rats, comparable to the clinical experiments mentioned above, or injection of CGRP into the trigeminal ganglion activated and sensitized a variety of dura-sensitive Aδ- and C-fibers and central trigeminovascular neurons in a delayed manner [141]. These findings support the general view that peripheral afferent input contributes to activation of central trigeminal neurons [142, 143]. Exogenous and endogenously released CGRP can reach CGRP receptors located on Schwann cells of nerve fibers in the periphery (e.g. the dura mater) and on cell bodies and satellite glial cells in the trigeminal ganglion, which is located outside the blood-brain barrier [96, 144]. Within the spinal trigeminal tract and nucleus, CGRP receptors may be reached by CGRP released locally from central afferent terminals [96, 98]. CGRP receptor-expressing neurons and CGRP-releasing neurons appear to represent partly distinct populations, suggesting that CGRP acts through paracrine rather than autoreceptor mechanisms. CGRP receptor activation increases cAMP and activates PKA-dependent signaling, which can modulate ion channels, enzymes, and transcriptional pathways [2]. One proposed mechanism is direct modulation of axonal excitability at nodes of Ranvier [145]. However, evidence for axonal CGRP receptors remains limited, and the functional consequences of such signaling are uncertain.

An alternative hypothesis is that CGRP acts primarily on glial elements, particularly Schwann cells and satellite glial cells, which then influence nearby afferents. Candidate downstream targets include purinergic receptors, COX-2, and iNOS, each of which could promote nociceptive signaling [146–149]. Because ERK-dependent transcriptional responses evolve over hours, this mechanism could help explain both delayed headache after CGRP infusion in migraineurs [36] and delayed afferent activation in preclinical models [141].

In addition to peripheral targets, central migraine-relevant mechanisms of CGRP deserve mention. CGRP injected into the posterior thalamus and medial cerebellar nuclei, as well as optogenetic activation of cerebellar CGRP neurons, has been shown to induce migraine-like behavior like photophobia in mice [150, 151]. These findings do not exclude the proposed peripheral endothelial–glial pathway but instead suggest that CGRP may contribute to migraine through multiple mechanisms operating at peripheral and central levels. While peripheral CGRP signaling may be particularly relevant for trigeminovascular nociception, central CGRP pathways may contribute to neurological and sensory symptoms associated with migraine. Additional support for a predominantly peripheral action of CGRP comes from comparisons with pituitary adenylate cyclase-activating polypeptide (PACAP). Although both CGRP and PACAP-38 induce headache in approximately two-thirds of susceptible individuals, premonitory symptoms—widely considered to reflect central nervous system involvement—occur much less frequently after CGRP infusion than after PACAP-38 administration [152]. This observation is consistent with, although not definitive proof of, a more peripheral site of action for CGRP.

## Role of Schwann cell activation in NO-TRPA1-CGRP signaling

**Fig. 4:**
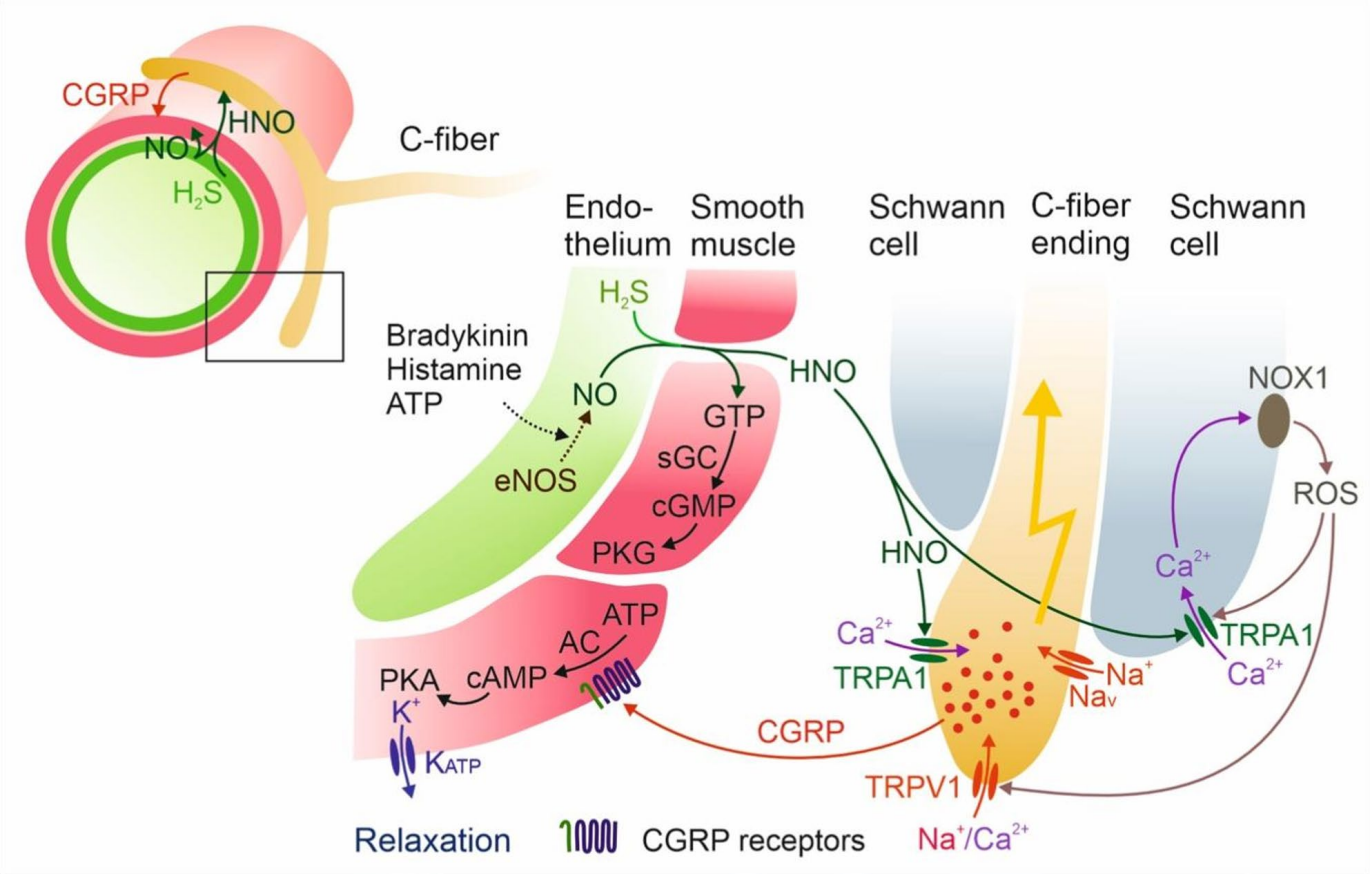
Activation of TRPA1 receptors on C-fiber axons and Schwann cells. Schwann cells may express NADPH oxidase (NOX1) to produce reactive oxygen species (ROS), which may signal back to activate TRPA1 and TRPV1. Axon depolarization may be sufficient to open voltage-gated sodium channels (Na_v_) and induce spiking (flash). Labeling, see Fig. 4

Schwann cells associated with perivascular afferents may express TRPA1 channels, which have been implicated in neuropathic pain and migraine-related behaviors [153–155]. TRPA1 immunoreactivity has been localized to Schwann cells of the rat sciatic nerve, and cultivated Schwann cells respond to the TRPA1 agonists allyl thioisocynate (AITC) with calcium inflow [156]. HNO released from arterial vessels may activate Schwann-cell TRPA1 channels and increase intracellular calcium levels (Fig. 4). Schwann cells can express NADPH oxidase 1 (NOX1), which is activated through intracellular calcium increase to produce reactive oxygen species (ROS) [153, 157]. ROS can activate TRPA1 and other TRP channels [158, 159] potentially amplifying signaling between Schwann cells and adjacent C-fibre axons and facilitating further CGRP release (Fig. 4). Activation of TRPA1 and TRPV1 channels may ultimately depolarize afferents sufficiently to generate action potentials.

As mentioned above, CGRP receptors are expressed in Schwann cells of trigeminal afferents, mainly at the nodes of Ranvier of Aδ-fibers [72, 96]. This distribution suggests that CGRP signaling may preferentially affect myelinated Aδ-fibers. Evidence for this hypothesis comes from recent experiments in rats, in which preferably meningeal Aδ-fibers and spinal trigeminal neurons with Aδ-fiber input were inhibited by a CGRP receptor antagonist [160] or after treatment with an anti-CGRP antibody [161].

**Fig. 5:**
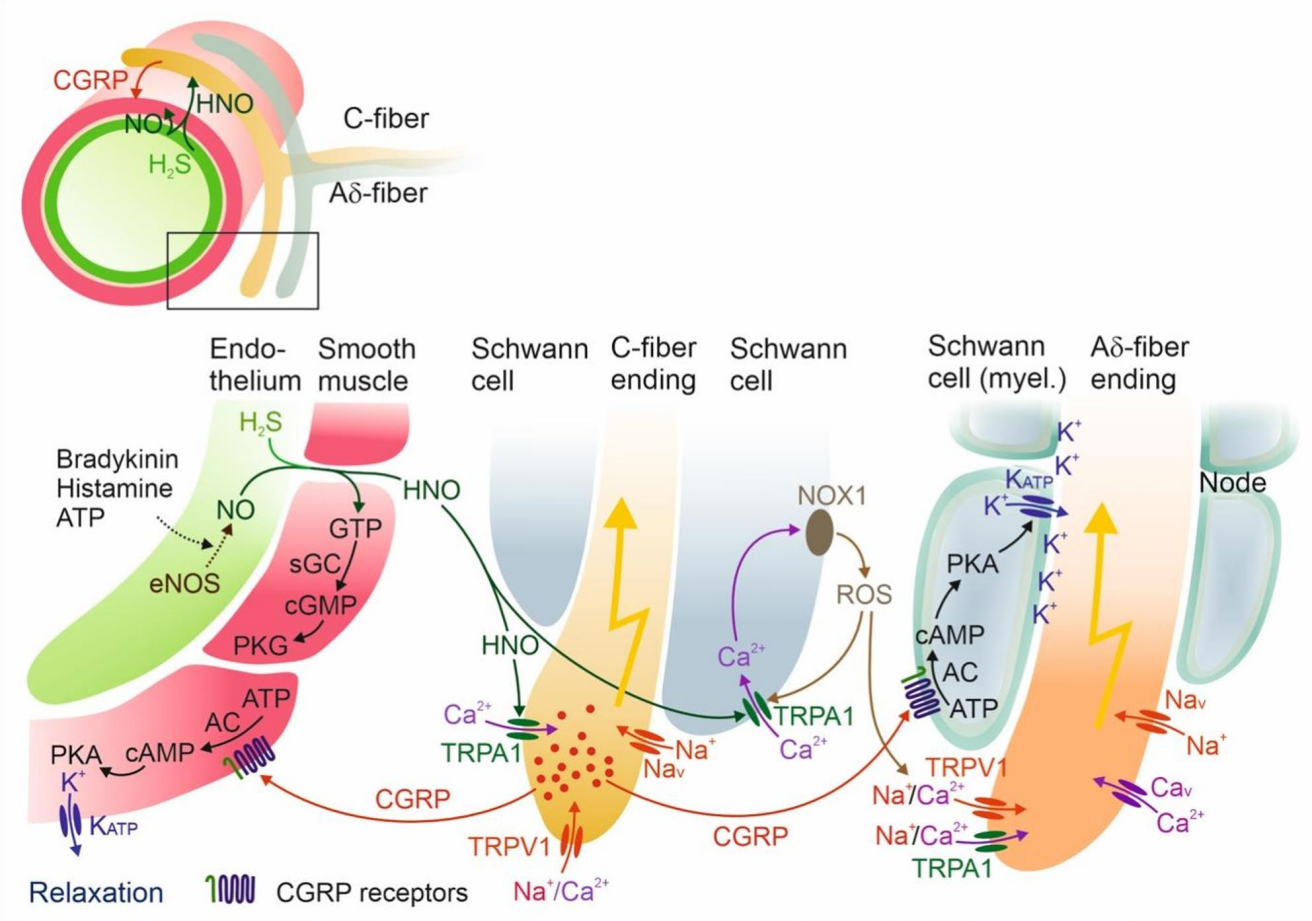
Possible CGRP signaling to myelinating Schwann cells inducing opening of ATP-sensitive potassium channels (K_ATP_) and accumulation of potassium ions in the intercellular space. In addition, ROS signaling to TRPV1 and TRPA1 receptor channels may directly activate Aδ-fibre axons, which produce action potentials (flash) when depolarization exceeds thresholds for voltage-gated calcium and sodium channels (Ca_v_, Na_v_). Labeling, see Fig. 5

Myelinating Schwann cells have also been shown to express subunits of ATP-sensitive potassium channels (K_ATP_) at paranodal sites located to Schmidt-Lanterman incisures [162]. Activation of these channels could alter local potassium homeostasis and thereby influence Aδ-fiber excitability (Fig. 5). This possibility is particularly interesting because K_ATP_ channel openers induce migraine-like responses in both rodent models and migraine patients [163–166]. However, selective activation of neuronal K_ATP_ channels does not appear sufficient to induce migraine-like pain, shifting attention toward vascular K_ATP_ channels and reinforcing a vascular contribution to migraine initiation [167]. Because myelinating Schwann cells can express TRPA1 [168], mediators such as HNO and ROS may influence both C-fibers and Aδ-fibers through these glial intermediaries [169] (see Fig. 5). Finally, trigeminal Aδ-afferents can also express TRPA1 and TRPV1 receptor channels [170] and may thus directly be activated by endogenous mediators like HNO and ROS (Fig. 5).

## Role of cortical spreading depression in CGRP signaling

Cortical spreading depression (CSD) can be induced experimentally by mechanical stimulation, potassium chloride, electrical current pulses, or optogenetic methods and can be monitored by electrophysiological or blood-flow measurements [171, 172]. CSD consists of a transient wave of neuronal depolarization followed by prolonged suppression of neuronal activity [172–174]. CSD spreads from the point of origin slowly over the cortex and is accompanied by variable and species-specific vascular responses, mostly consisting of short arterial vasoconstriction followed by fluctuating vasodilatation [175, 176].

CSD is widely regarded as the physiological substrate of migraine aura, a concept supported by extensive experimental and clinical evidence [173, 177–180]. A recent intracranial recording study provided the first direct human confirmation of cortical spreading depression during migraine aura [181]. During stereotactic electroencephalographic monitoring for pre-surgical epilepsy evaluation, a propagating wave of low-voltage cortical suppression was captured as it spread from the occipital cortex, temporally corresponding to the patient’s visual aura and subsequent headache [181]. The finding directly confirms a mechanism that has remained central to migraine pathophysiology since Leão’s seminal description of cortical spreading depression in 1944 [182]. This observation is further supported by the characteristic biphasic cerebral blood flow response during migraine aura, first demonstrated by early imaging studies, which has long been interpreted as a vascular correlate of cortical spreading depression [183, 184]. Notably, headache may begin during the phase of reduced cortical blood flow, arguing against a purely vasodilatory explanation of migraine pain [185]. At the same time, the ability of vasodilatory substances to trigger migraine-like headache continues to support an important vascular contribution [166].

How CSD leads to meningeal nociception remains incompletely understood. CSD has been shown to promote activation and sensitization of meningeal afferents and spinal trigeminal neurons with meningeal afferent input in rats [186–188]. These effects occur with delays ranging from minutes to approximately one hour, consistent with indirect signaling mechanisms [186–189]. Several studies have linked CSD and CGRP signaling. CSD may trigger CGRP release from perivascular afferents [190], whereas other studies suggest ROS-TRPA1-CGRP signaling may contribute to CSD initiation [191]. CGRP receptor antagonists did not change CSD in rats [192] but attenuated CSD-induced nociceptive behavior and signaling in the spinal trigeminal nucleus [193]. Taken together, these findings suggest that CGRP may not be essential for CSD generation but may contribute to downstream nociceptive consequences of CSD. Within our proposed framework, CSD-associated vascular changes could promote NO-, HNO-, and ROS-dependent signaling, thereby facilitating activation of trigeminovascular afferents [194, 195]. This concept shares similarities with reversible cerebral vasoconstriction syndrome (RCVS), another disorder characterized by dysregulated vascular tone and severe headache [196, 197]. We speculate that activation may begin at cortical vessels and subsequently engages meningeal and ganglionic signaling pathways [198]. However, whether this occurs through afferent collaterals, intraganglionic communication, or other mechanisms remains unknown and requires direct experimental investigation.

### Possible role of NO-TRPA1-CGRP signaling the trigeminal ganglion

**Fig. 6:**
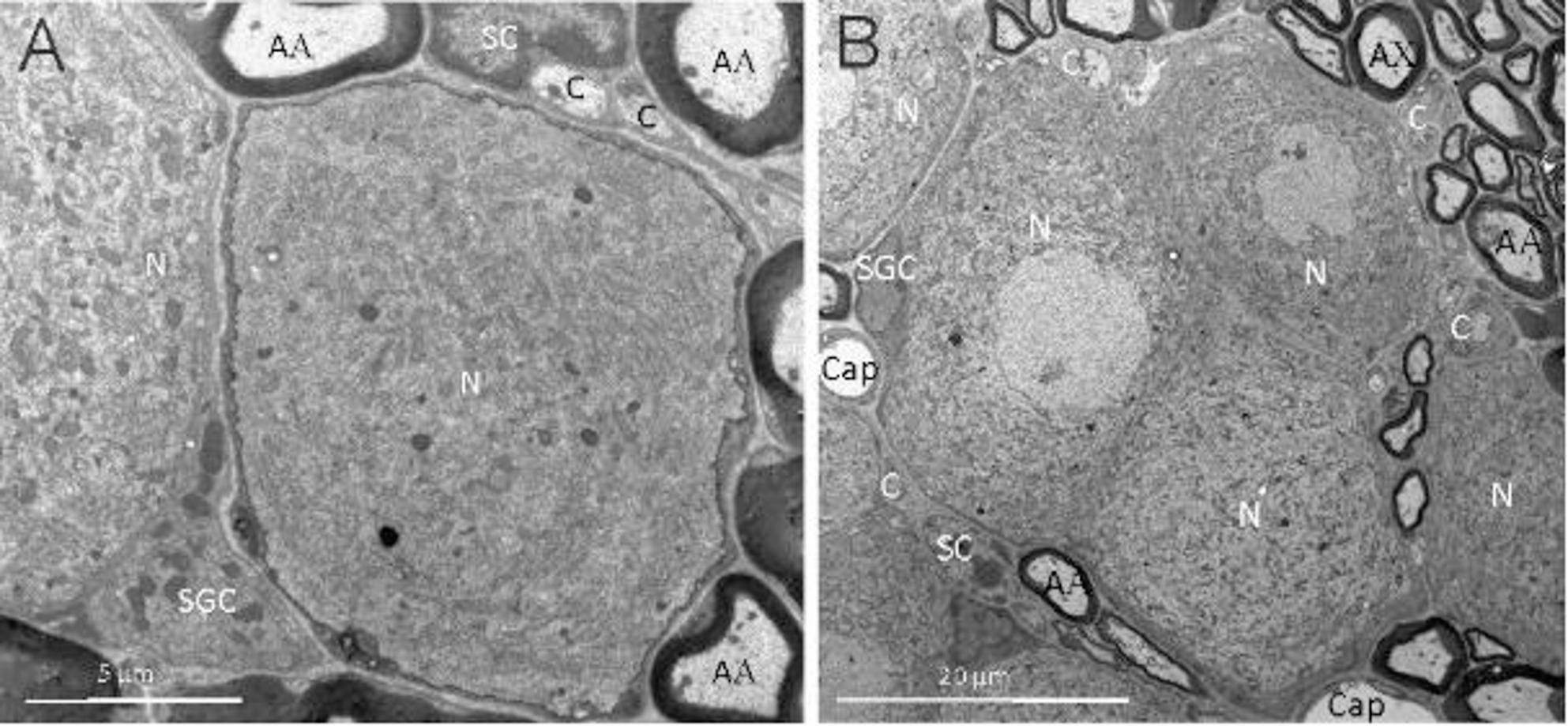
Electron microscopic images of rat trigeminal ganglion cells. **(A)** Satellite glial cells (SGC) surround neuronal cell bodies (N) tightly, isolating them from other neurons, from myelinated Aδ-fibre axons (AA) and Schwann cells (SC) with C-fibre axons (C). **(B)** C-fibre neuronal cell bodies can form clusters, in which neurons are separated only by very thin extension of satellite glial cells; Cap, capillary. Courtesy of A. Hilpert and W. Neuhuber

**Fig. 7:**
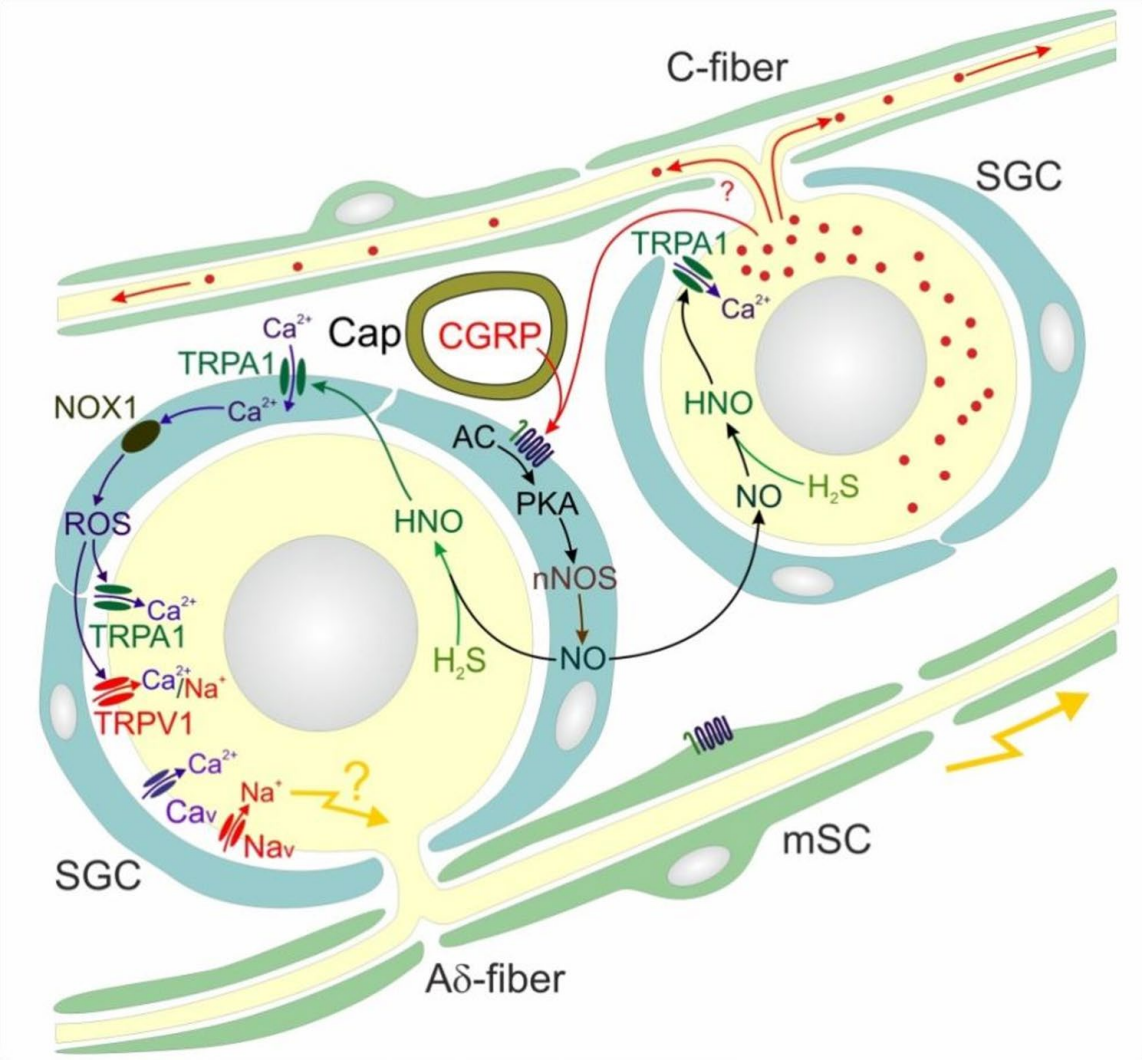
Hypothetic CGRP signaling in the trigeminal ganglion. Circulating (e.g., through experimental infusion via capillaries, Cap) or locally released CGRP (red arrows) reaches satellite glial cells (SGC), binds to CGRP receptors and activates NO generation, which together with H_2_S forms nitroxyl (HNO). HNO activates TRPA1 receptor channels of SGC followed by calcium inflow, which activates the NADPH oxidase 1 (NOX1) to generate reactive oxygen species (ROS). ROS activate TRPA1 and TRPV1 receptor channels of Aδ-fiber neurons, and upon depolarization may open voltage-gated calcium and sodium channels possibly generating action potentials (flash). NO-HNO-TRPA1 signaling to adjacent neurons can potentially also initiate CGRP release from peptidergic C-fibre neurons, closing the loop of CGRP-NO-HNO-TRPA1-ROS signaling. CGRP receptors may also be located on myelinating Schwann cells (mSC). Further labeling, see Fig. 4

The trigeminal ganglion is located outside the blood-brain barrier and is therefore accessible to circulating mediators [144]. CGRP has been shown to be released from isolated trigeminal ganglia in various experiments [14, 199–202]. Small trigeminal neurons frequently express CGRP, whereas satellite glial cells, Schwann cells, and subsets of neurons express CGRP receptors [96, 203–205]. Most neuronal cell bodies are tightly surrounded by satellite glial cells (Fig. 6A), suggesting that glial elements may serve as important intermediaries for circulating CGRP [206]. Neuronal clusters separated by thin glial layers may facilitate local intercellular signaling (Fig. 6B). We therefore propose that CGRP released from C-fibre neurons acts on neighbouring satellite glial cells and receptor-expressing neurons [207]. Similar to Schwann cells, satellite glial cells may generate ROS, which could enhance neuronal excitability through TRP-channel activation (Fig. 7).

In cultured trigeminal glial cells, CGRP stimulates iNOS expression and NO production [203, 208]. Subsets of trigeminal neurons express nNOS, TRPA1, and enzymes involved in H_2_S production, providing the molecular machinery for HNO-mediated signaling similar to that proposed in the meninges [209](Fig. 7). Neuronal depolarization can also release ATP [210] potentially engaging both glial P2Y receptors and neuronal P2X receptors. Interestingly, CGRP has been shown in cultured trigeminal ganglion cells to upregulate P2X receptor expression [136]. Whether this occurs in the intact ganglion remains uncertain. Recent evidence suggests that the trigeminal ganglion is accessible not only from the circulation but also from the cerebrospinal fluid [211]. Consequently, circulating CGRP [49], locally released CGRP, and other soluble mediators may gain access to ganglionic structures and influence neuronal or glial activity, and noxious substances may be washed into the ganglion and activate neurons directly (Fig. 7).

## CGRP signaling within the spinal trigeminal nucleus

At the spinal level, CGRP released from primary afferent terminals acts as a neuromodulator that facilitates synaptic transmission [101, 212–215]. Numerous CGRP-containing fibers are present within the trigeminal tract and superficial laminae of the spinal trigeminal nucleus [96]. Possibly, recently detected spinal trigeminal interneurons may also come into account as sources of CGRP, although they respond rather to low-threshold than noxious stimuli [216]. In contrast, CGRP receptor components are predominantly localized to fibers and glomerular structures rather than neuronal somata [96, 98]. Immunostaining for CLR and RAMP1 is concentrated in fibrous and granular structures within laminae II–III and is largely absent from neuronal cell bodies, supporting a predominantly presynaptic role of CGRP signaling [96, 217].

Using slices of the mouse medulla, whole-cell patch clamp recordings of superficial spinal trigeminal nucleus neurons confirmed the modulatory role of CGRP [101]. CGRP lowered activation thresholds and increased neuronal firing, findings consistent with facilitation of nociceptive transmission [101]. However, iontophoretic application of CGRP excites second-order neurons, while CGRP receptor blockade reduces glutamate-evoked activity, suggesting that postsynaptic mechanisms may also contribute [218].

Further evidence for a spinal modulatory role of CGRP comes from animal studies showing that systemic administration of the CGRP receptor antagonist olcegepant reduces trigeminal neuronal activity and inhibits capsaicin-induced Fos expression [219, 220]. In contrast, local administration of olcegepant to the dura mater or the trigeminal ganglion was widely ineffective [219, 221], underscoring a facilitating action of endogenous CGRP at central sites. Overall, the relative contributions of peripheral, ganglionic, and spinal CGRP signaling remain incompletely understood.

## CGRP signaling to immune-derived cells

CGRP via CGRP receptors signal also to immune cells, which in the trigeminovascular system is especially relevant in situations of meningeal infections [222]. Mononuclear cells expressing CGRP receptor components in the dura mater likely include resident and infiltrating macrophages [96]. Such observations suggest that CGRP can influence the meningeal immune system; this topic has been comprehensively reviewed elsewhere [40, 223–225]. Activation of immune cells by CGRP may contribute to headache generation through release of cytokines, prostaglandins and other inflammatory mediators [226]. In rat dura mater, COX-1 is primarily associated with vascular endothelium and mast cells, whereas COX-2 is mainly expressed by resident macrophages [227].

CGRP can also promote histamine release from dural mast cells, thereby facilitating vasodilatation [137, 228–230]. Meningeal mast cells are closely associated with blood vessels and perivascular nerve bundles [231–233]. CGRP receptor expression has been reported in rodent meninges [96] but confirmation in human meninges remains limited [72]. Experimental exposure of rat dura mater to CGRP increases histamine release [137, 228, 230]. Although mast-cell activation remains a proposed component of neurogenic inflammation, its precise contribution to migraine pathophysiology remains debated [234–236].

## Conclusions

CGRP, either exogenously applied or endogenously released, has a strong vasodilatory effect on arterial blood vessels in the trigeminovascular system. Whether vasodilatation directly contributes to activation of perivascular afferents, for example through ATP release and P2X receptor activation, remains uncertain. Current evidence suggests that many CGRP effects may occur indirectly through Schwann cells in the periphery and satellite glial cells within the trigeminal ganglion. These glial responses may include induction of pro-nociceptive mediators such as nitric oxide species. Such transcription-dependent mechanisms provide a plausible explanation for the delayed onset of migraine-like headache following CGRP exposure. Within this framework, as a first step in the cascade of nociceptive events releasing spontaneous migraine attacks, NO and HNO signaling may activate TRPA1 channels, while purinergic pathways may further amplify afferent excitation. Potential initiating events include endothelial dysfunction, inflammation, oxidative stress, and cortical spreading depression, although direct evidence remains limited. The unique enrichment of CGRP-containing afferents within the trigeminovascular system may help explain why these mechanisms preferentially generate headache rather than pain in other tissues.

The contrasting therapeutic experience with CGRP- and NOS-directed interventions is noteworthy [51], but the limited success of NOS-targeted therapies should not be interpreted as evidence against a central role of NO in migraine. In fact, the efficacy of non-selective NOS inhibition in both experimental models and clinical studies [237–239] suggests that NO signaling remains a fundamental component of migraine biology. We therefore propose that neuronal and endothelial NOS occupy a pivotal position within the TRPA1–NOS–CGRP signaling cascade, linking vascular, glial, and neuronal mechanisms that ultimately culminate in migraine pain.

Taken together, we propose a testable hypothesis, in which CGRP initiates a delayed endothelial–glial signaling cascade that ultimately sensitizes trigeminovascular afferents. Endothelial signaling is proposed to represent the initiating event, while ganglionic and spinal glial mechanisms are proposed to amplify and sustain trigeminovascular sensitization. Individual components of this cascade are supported by varying levels of experimental evidence and remain subject to future experimental validation. While several elements of this model remain provisional, it integrates vascular, glial, ganglionic, and trigeminal observations into a unified framework and identifies experimentally tractable pathways for future investigation.

## Supplementary Information

Below is the link to the electronic supplementary material.

Supplementary Material 1

## Data Availability

No datasets were generated or analysed during the current study.

## References

[1] Amara SG, Jonas V, Rosenfeld MG et al (1982) Alternative RNA processing in calcitonin gene expression generates mRNAs encoding different polypeptide products. Nature 298:240–244 6283379 10.1038/298240a0

[2] Benarroch EE (2011) CGRP: sensory neuropeptide with multiple neurologic implications. Neurology 77:281–287. 10.1212/WNL.0b013e31822550e2 21768598 10.1212/WNL.0b013e31822550e2

[3] Holzer P, Barthó L, Matusák O, Bauer V (1989) Calcitonin gene-related peptide action on intestinal circular muscle. Am J Physiol 256:G546–552 2564254 10.1152/ajpgi.1989.256.3.G546

[4] Juaneda C, Dumont Y, Quirion R (2000) The molecular pharmacology of CGRP and related peptide receptor subtypes. Trends Pharmacol Sci 21:432–438 11121574 10.1016/s0165-6147(00)01555-8

[5] Russell FA, King R, Smillie S-J et al (2014) Calcitonin gene-related peptide: physiology and pathophysiology. Physiol Rev 94:1099–1142. 10.1152/physrev.00034.2013 25287861 10.1152/physrev.00034.2013 PMC4187032

[6] Iyengar S, Ossipov MH, Johnson KW (2017) The role of calcitonin gene-related peptide in peripheral and central pain mechanisms including migraine. Pain 158:543–559. 10.1097/j.pain.0000000000000831 28301400 10.1097/j.pain.0000000000000831 PMC5359791

[7] Kessler F, Habelt C, Averbeck B et al (1999) Heat-induced release of CGRP from isolated rat skin and effects of bradykinin and the protein kinase C activator PMA. Pain 83:289–295. 10.1016/s0304-3959(99)00108-6 10534601 10.1016/s0304-3959(99)00108-6

[8] Hoffmann J, Wecker S, Neeb L et al (2012) Primary trigeminal afferents are the main source for stimulus-induced CGRP release into jugular vein blood and CSF. Cephalalgia Int J Headache 32:659–667. 10.1177/0333102412447701 10.1177/0333102412447701 22652383

[9] Kageneck C, Nixdorf-Bergweiler BE, Messlinger K, Fischer MJ (2014) Release of CGRP from mouse brainstem slices indicates central inhibitory effect of triptans and kynurenate. J Headache Pain 15:7. 10.1186/1129-2377-15-7 24506953 10.1186/1129-2377-15-7 PMC3922191

[10] Dux M, Will C, Eberhardt M et al (2017) Stimulation of rat cranial dura mater with potassium chloride causes CGRP release into the cerebrospinal fluid and increases medullary blood flow. Neuropeptides 64:61–68. 10.1016/j.npep.2017.02.080 28202186 10.1016/j.npep.2017.02.080

[11] Mason RT, Peterfreund RA, Sawchenko PE et al (1984) Release of the predicted calcitonin gene-related peptide from cultured rat trigeminal ganglion cells. Nature 308:653–655. 10.1038/308653a0 6369148 10.1038/308653a0

[12] Sanada M, Yasuda H, Omatsu-Kanbe M et al (2002) Increase in intracellular Ca(2+) and calcitonin gene-related peptide release through metabotropic P2Y receptors in rat dorsal root ganglion neurons. Neuroscience 111:413–422. 10.1016/s0306-4522(02)00005-2 11983326 10.1016/s0306-4522(02)00005-2

[13] Durham PL, Masterson CG (2013) Two Mechanisms Involved in Trigeminal CGRP Release: Implications for Migraine Treatment. Headache J Head Face Pain 53:67–80. 10.1111/j.1526-4610.2012.02262.x 10.1111/j.1526-4610.2012.02262.x PMC3540191 23095108

[14] Kleeberg-Hartmann J, Vogler B, Messlinger K (2021) Petasin and isopetasin reduce CGRP release from trigeminal afferents indicating an inhibitory effect on TRPA1 and TRPV1 receptor channels. J Headache Pain 22:23. 10.1186/s10194-021-01235-5 33849430 10.1186/s10194-021-01235-5 PMC8042690

[15] Eberhardt M, Stueber T, de la Roche J et al (2017) TRPA1 and TRPV1 are required for lidocaine-evoked calcium influx and neuropeptide release but not cytotoxicity in mouse sensory neurons. PLoS ONE 12:e0188008. 10.1371/journal.pone.0188008 29141003 10.1371/journal.pone.0188008 PMC5687772

[16] Gebhardt LA, Kichko TI, Fischer MJM, Reeh PW (2020) TRPA1-dependent calcium transients and CGRP release in DRG neurons require extracellular calcium. J Cell Biol 219:e201702151. 10.1083/jcb.201702151 32434221 10.1083/jcb.201702151 PMC7265312

[17] Franco-Cereceda A, Henke H, Lundberg JM et al (1987) Calcitonin gene-related peptide (CGRP) in capsaicin-sensitive substance P-immunoreactive sensory neurons in animals and man: distribution and release by capsaicin. Peptides 8:399–410. 10.1016/0196-9781(87)90117-3 2438668 10.1016/0196-9781(87)90117-3

[18] Bellamy JL, Cady RK, Durham PL (2006) Salivary levels of CGRP and VIP in rhinosinusitis and migraine patients. Headache 46:24–33. 10.1111/j.1526-4610.2006.00294.x 16412148 10.1111/j.1526-4610.2006.00294.x

[19] Iyengar S, Johnson KW, Ossipov MH, Aurora SK (2019) CGRP and the Trigeminal System in Migraine. Headache 59:659–681. 10.1111/head.13529 30982963 10.1111/head.13529 PMC6593989

[20] Kamm K, Straube A, Ruscheweyh R (2019) Calcitonin gene-related peptide levels in tear fluid are elevated in migraine patients compared to healthy controls. Cephalalgia Int J Headache 39:1535–1543. 10.1177/0333102419856640 10.1177/0333102419856640 31603037

[21] Messlinger K, Vogler B, Kuhn A et al (2021) CGRP measurements in human plasma - a methodological study. Cephalalgia Int J Headache 41:1359–1373. 10.1177/03331024211024161 10.1177/03331024211024161 PMC8592105 34266288

[22] Pedersen-Bjergaard U, Nielsen LB, Jensen K et al (1991) Calcitonin gene-related peptide, neurokinin A and substance P: effects on nociception and neurogenic inflammation in human skin and temporal muscle. Peptides 12:333–337. 10.1016/0196-9781(91)90022-h 1712469 10.1016/0196-9781(91)90022-h

[23] Sann H, Friedrich R, Pierau FK (1996) Substance P and calcitonin gene-related peptide in the chicken skin: distribution and cardiovascular effects. Neuropeptides 30:273–281. 10.1016/s0143-4179(96)90073-6 8819151 10.1016/s0143-4179(96)90073-6

[24] Edvinsson JC, Reducha PV, Sheykhzade M et al (2021) Neurokinins and their receptors in the rat trigeminal system: Differential localization and release with implications for migraine pain. Mol Pain 17:17448069211059400. 10.1177/17448069211059400 34898306 10.1177/17448069211059400 PMC8679402

[25] Holzer P (1998) Neurogenic vasodilatation and plasma leakage in the skin. Gen Pharmacol 30:5–11. 10.1016/s0306-3623(97)00078-5 9457475 10.1016/s0306-3623(97)00078-5

[26] Samsam M, Coveñas R, Ahangari R et al (2000) Simultaneous depletion of neurokinin A, substance P and calcitonin gene-related peptide from the caudal trigeminal nucleus of the rat during electrical stimulation of the trigeminal ganglion. Pain 84:389–395. 10.1016/s0304-3959(99)00240-7 10666545 10.1016/s0304-3959(99)00240-7

[27] Foreman JC (1988) The skin as an organ for the study of the pharmacology of neuropeptides. Skin Pharmacol Off J Skin Pharmacol Soc 1:77–83. 10.1159/000210752 10.1159/000210752 3078645

[28] Schmelz M, Luz O, Averbeck B, Bickel A (1997) Plasma extravasation and neuropeptide release in human skin as measured by intradermal microdialysis. Neurosci Lett 230:117–120. 10.1016/s0304-3940(97)00494-1 9259478 10.1016/s0304-3940(97)00494-1

[29] Wallengren J, Wang ZY (1993) Interaction between tachykinins and CGRP in human skin. Acta Derm Venereol 73:259–261. 10.2340/0001555573259261 7506467 10.2340/0001555573259261

[30] Weidner C, Klede M, Rukwied R et al (2000) Acute effects of substance P and calcitonin gene-related peptide in human skin–a microdialysis study. J Invest Dermatol 115:1015–1020. 10.1046/j.1523-1747.2000.00142.x 11121135 10.1046/j.1523-1747.2000.00142.x

[31] Fuller RW, Conradson TB, Dixon CM et al (1987) Sensory neuropeptide effects in human skin. Br J Pharmacol 92:781–788. 10.1111/j.1476-5381.1987.tb11381.x 2892555 10.1111/j.1476-5381.1987.tb11381.x PMC1853716

[32] Sauerstein K, Klede M, Hilliges M, Schmelz M (2000) Electrically evoked neuropeptide release and neurogenic inflammation differ between rat and human skin. J Physiol 529 Pt 3:803–810. 10.1111/j.1469-7793.2000.00803.x 10.1111/j.1469-7793.2000.00803.x PMC2270217 11118507

[33] Goadsby PJ (2009) The vascular theory of migraine–a great story wrecked by the facts. Brain J Neurol 132:6–7. 10.1093/brain/awn321 10.1093/brain/awn321 19098031

[34] Goadsby PJ, Edvinsson L (1993) The trigeminovascular system and migraine: studies characterizing cerebrovascular and neuropeptide changes seen in humans and cats. Ann Neurol 33:48–56. 10.1002/ana.410330109 8388188 10.1002/ana.410330109

[35] Alpuente A, Gallardo VJ, Asskour L et al (2022) Salivary CGRP can monitor the different migraine phases: CGRP (in)dependent attacks. Cephalalgia Int J Headache 42:186–196. 10.1177/03331024211040467 10.1177/03331024211040467 34601944

[36] Lassen LH, Haderslev PA, Jacobsen VB et al (2002) CGRP may play a causative role in migraine. Cephalalgia Int J Headache 22:54–61 10.1046/j.1468-2982.2002.00310.x 11993614

[37] Hansen JM, Hauge AW, Olesen J, Ashina M (2010) Calcitonin gene-related peptide triggers migraine-like attacks in patients with migraine with aura. Cephalalgia Int J Headache 30:1179–1186. 10.1177/0333102410368444 10.1177/0333102410368444 20855363

[38] Vollesen ALH, Snoer A, Beske RP et al (2018) Effect of Infusion of Calcitonin Gene-Related Peptide on Cluster Headache Attacks: A Randomized Clinical Trial. JAMA Neurol 75:1187–1197. 10.1001/jamaneurol.2018.1675 29987329 10.1001/jamaneurol.2018.1675 PMC6233850

[39] Iyengar S, Johnson KW, Ossipov MH, Aurora SK (2019) CGRP and the Trigeminal System in Migraine. Headache 59:659–681. 10.1111/head.13529 30982963 10.1111/head.13529 PMC6593989

[40] Russo AF, Hay DL (2023) CGRP physiology, pharmacology, and therapeutic targets: migraine and beyond. Physiol Rev 103:1565–1644. 10.1152/physrev.00059.2021 36454715 10.1152/physrev.00059.2021 PMC9988538

[41] Edvinsson L, Haanes KA, Warfvinge K, Krause DN (2018) CGRP as the target of new migraine therapies - successful translation from bench to clinic. Nat Rev Neurol 14:338–350. 10.1038/s41582-018-0003-1 29691490 10.1038/s41582-018-0003-1

[42] Wattiez A-S, Sowers LP, Russo AF (2020) Calcitonin gene-related peptide (CGRP): role in migraine pathophysiology and therapeutic targeting. Expert Opin Ther Targets 24:91–100. 10.1080/14728222.2020.1724285 32003253 10.1080/14728222.2020.1724285 PMC7050542

[43] Caronna E, Alpuente A, Torres-Ferrus M, Pozo-Rosich P (2024) CGRP monoclonal antibodies and CGRP receptor antagonists (Gepants) in migraine prevention. Handb Clin Neurol 199:107–124. 10.1016/B978-0-12-823357-3.00024-0 38307640 10.1016/B978-0-12-823357-3.00024-0

[44] Goadsby PJ, Edvinsson L (1994) Human in vivo evidence for trigeminovascular activation in cluster headache. Neuropeptide changes and effects of acute attacks therapies. Brain J Neurol 117(Pt 3):427–434 10.1093/brain/117.3.427 7518321

[45] Goadsby PJ, Hargreaves RJ (2000) Mechanisms of action of serotonin 5-HT1B/D agonists: insights into migraine pathophysiology using rizatriptan. Neurology 55:S8–14 11089513

[46] Eltorp CT, Jansen-Olesen I, Hansen AJ (2000) Release of calcitonin gene-related peptide (CGRP) from guinea pig dura mater in vitro is inhibited by sumatriptan but unaffected by nitric oxide. Cephalalgia Int J Headache 20:838–844 10.1046/j.1468-2982.2000.00131.x 11167915

[47] Gupta S, Amrutkar DV, Mataji A et al (2010) Evidence for CGRP re-uptake in rat dura mater encephali. Br J Pharmacol 161:1885–1898. 10.1111/j.1476-5381.2010.01012.x 20804493 10.1111/j.1476-5381.2010.01012.x PMC3010590

[48] Marics B, Peitl B, Pázmándi K et al (2017) Diet-Induced Obesity Enhances TRPV1-Mediated Neurovascular Reactions in the Dura Mater. Headache 57:441–454. 10.1111/head.13033 28133727 10.1111/head.13033

[49] Risch M, Vogler B, Dux M, Messlinger K (2021) CGRP outflow into jugular blood and cerebrospinal fluid and permeance for CGRP of rat dura mater. J Headache Pain 22:105. 10.1186/s10194-021-01320-9 34496764 10.1186/s10194-021-01320-9 PMC8424805

[50] Rasmussen RH, Jansen-Olesen I, Kristensen DM, Christensen SL (2022) Ex vivo Release of Calcitonin Gene-Related Peptide from the Trigeminovascular System in Rodents. J Vis Exp JoVE. 10.3791/63723 35635478 10.3791/63723

[51] Dux M, Messlinger K (2025) Substance P release from rat dura mater is inversely correlated with CGRP release- experiments using glycerol trinitrate and anti-CGRP antibodies. J Headache Pain 26:119. 10.1186/s10194-025-02050-y 40380328 10.1186/s10194-025-02050-y PMC12085035

[52] Avona A, Burgos-Vega C, Burton MD et al (2019) Dural Calcitonin Gene-Related Peptide Produces Female-Specific Responses in Rodent Migraine Models. J Neurosci Off J Soc Neurosci 39:4323–4331. 10.1523/JNEUROSCI.0364-19.2019 10.1523/JNEUROSCI.0364-19.2019 PMC6538861 30962278

[53] Levy D, Burstein R, Strassman AM (2005) Calcitonin gene-related peptide does not excite or sensitize meningeal nociceptors: implications for the pathophysiology of migraine. Ann Neurol 58:698–705. 10.1002/ana.20619 16240341 10.1002/ana.20619

[54] Al-Karagholi MA-M, Kalatharan V, Fagerberg PS, Amin FM (2023) The vascular role of CGRP: a systematic review of human studies. Front Neurol 14:1204734. 10.3389/fneur.2023.1204734 37483452 10.3389/fneur.2023.1204734 PMC10359159

[55] Egea SC, Dickerson IM (2012) Direct interactions between calcitonin-like receptor (CLR) and CGRP-receptor component protein (RCP) regulate CGRP receptor signaling. Endocrinology 153:1850–1860. 10.1210/en.2011-1459 22315449 10.1210/en.2011-1459 PMC3320266

[56] Hay DL, Garelja ML, Poyner DR, Walker CS (2018) Update on the pharmacology of calcitonin/CGRP family of peptides: IUPHAR Review 25. Br J Pharmacol 175:3–17. 10.1111/bph.14075 29059473 10.1111/bph.14075 PMC5740251

[57] Hendrikse ER, Bower RL, Hay DL, Walker CS (2019) Molecular studies of CGRP and the CGRP family of peptides in the central nervous system. Cephalalgia 39:403–419. 10.1177/0333102418765787 29566540 10.1177/0333102418765787

[58] Walker CS, Eftekhari S, Bower RL et al (2015) A second trigeminal CGRP receptor: function and expression of the AMY1 receptor. Ann Clin Transl Neurol 2:595–608. 10.1002/acn3.197 26125036 10.1002/acn3.197 PMC4479521

[59] Russo AF, Hay DL (2023) CGRP physiology, pharmacology, and therapeutic targets: migraine and beyond. Physiol Rev 103:1565–1644. 10.1152/physrev.00059.2021 36454715 10.1152/physrev.00059.2021 PMC9988538

[60] Miyoshi H, Nakaya Y (1995) Calcitonin gene-related peptide activates the K+ channels of vascular smooth muscle cells via adenylate cyclase. Basic Res Cardiol 90:332–336. 10.1007/BF00797911 8534258 10.1007/BF00797911

[61] Kleppisch T, Nelson MT (1995) ATP-sensitive K+ currents in cerebral arterial smooth muscle: pharmacological and hormonal modulation. Am J Physiol 269:H1634–1640. 10.1152/ajpheart.1995.269.5.H1634 7503259 10.1152/ajpheart.1995.269.5.H1634

[62] Brian JE, Faraci FM, Heistad DD (1996) Recent insights into the regulation of cerebral circulation. Clin Exp Pharmacol Physiol 23:449–457 8800565 10.1111/j.1440-1681.1996.tb02760.x

[63] Permpoonputtana K, Porter JE, Govitrapong P (2016) Calcitonin gene-related peptide mediates an inflammatory response in Schwann cells via cAMP-dependent ERK signaling cascade. Life Sci 144:19–25. 10.1016/j.lfs.2015.11.015 26596264 10.1016/j.lfs.2015.11.015

[64] Yu X-J, Li C-Y, Wang K-Y, Dai H-Y (2006) Calcitonin gene-related peptide regulates the expression of vascular endothelial growth factor in human HaCaT keratinocytes by activation of ERK1/2 MAPK. Regul Pept 137:134–139. 10.1016/j.regpep.2006.07.001 16904202 10.1016/j.regpep.2006.07.001

[65] Walker CS, Raddant AC, Woolley MJ et al (2018) CGRP receptor antagonist activity of olcegepant depends on the signalling pathway measured. Cephalalgia Int J Headache 38:437–451. 10.1177/0333102417691762 10.1177/0333102417691762 PMC5494006 28165287

[66] Edvinsson L (2017) The Trigeminovascular Pathway: Role of CGRP and CGRP Receptors in Migraine. Headache 57 Suppl 2:47–55. 10.1111/head.13081 10.1111/head.13081 28485848

[67] Uddman R, Edvinsson L, Jansen I et al (1986) Peptide-containing nerve fibres in human extracranial tissue: a morphological basis for neuropeptide involvement in extracranial pain? Pain 27:391–399. 10.1016/0304-3959(86)90162-4 2433670 10.1016/0304-3959(86)90162-4

[68] Kosaras B, Jakubowski M, Kainz V, Burstein R (2009) Sensory innervation of the calvarial bones of the mouse. J Comp Neurol 515:331–348. 10.1002/cne.22049 19425099 10.1002/cne.22049 PMC2710390

[69] Schueler M, Messlinger K, Dux M et al (2013) Extracranial projections of meningeal afferents and their impact on meningeal nociception and headache. Pain 154:1622–1631. 10.1016/j.pain.2013.04.040 23707274 10.1016/j.pain.2013.04.040

[70] Andres KH, von Düring M, Muszynski K, Schmidt RF (1987) Nerve fibres and their terminals of the dura mater encephali of the rat. Anat Embryol (Berl) 175:289–301 3826655 10.1007/BF00309843

[71] Messlinger K, Hanesch U, Baumgärtel M et al (1993) Innervation of the dura mater encephali of cat and rat: ultrastructure and calcitonin gene-related peptide-like and substance P-like immunoreactivity. Anat Embryol (Berl) 188:219–237. 10.1007/BF00188214 7504417 10.1007/BF00188214

[72] Eftekhari S, Warfvinge K, Blixt FW, Edvinsson L (2013) Differentiation of nerve fibers storing CGRP and CGRP receptors in the peripheral trigeminovascular system. J Pain Off J Am Pain Soc 14:1289–1303. 10.1016/j.jpain.2013.03.010 10.1016/j.jpain.2013.03.010 23958278

[73] Schueler M, Neuhuber WL, De Col R, Messlinger K (2014) Innervation of rat and human dura mater and pericranial tissues in the parieto-temporal region by meningeal afferents. Headache 54:996–1009. 10.1111/head.12371 24673461 10.1111/head.12371

[74] Jacquin MF, Chiaia NL, Rhoades RW (1990) Trigeminal projections to contralateral dorsal horn: central extent, peripheral origins, and plasticity. Somatosens Mot Res 7:153–183. 10.3109/08990229009144705 2378191 10.3109/08990229009144705

[75] Shigenaga Y, Okamoto T, Nishimori T et al (1986) Oral and facial representation in the trigeminal principal and rostral spinal nuclei of the cat. J Comp Neurol 244:1–18. 10.1002/cne.902440102 3950088 10.1002/cne.902440102

[76] Phelan KD, Falls WM (1989) The interstitial system of the spinal trigeminal tract in the rat: anatomical evidence for morphological and functional heterogeneity. Somatosens Mot Res 6:367–399 2547273 10.3109/08990228909144682

[77] Sugimoto T, Fujiyoshi Y, Xiao C et al (1997) Central projection of calcitonin gene-related peptide (CGRP)- and substance P (SP)-immunoreactive trigeminal primary neurons in the rat. J Comp Neurol 378(19970217):425–442. 10.1002/(sici)1096-9861)378:3<425::aid-cne9>3.0.co;2-5. 10.1002/(sici)1096-9861(19970217)378:3<425::aid-cne9>3.0.co;2-5 9034901

[78] Wang H, Wei F, Dubner R, Ren K (2006) Selective distribution and function of primary afferent nociceptive inputs from deep muscle tissue to the brainstem trigeminal transition zone. J Comp Neurol 498:390–402. 10.1002/cne.21062 16871539 10.1002/cne.21062

[79] Liu Y, Broman J, Edvinsson L (2008) Central projections of the sensory innervation of the rat middle meningeal artery. Brain Res 1208:103–110. 10.1016/j.brainres.2008.02.078 18395192 10.1016/j.brainres.2008.02.078

[80] O’Connor TP, van der Kooy D (1988) Enrichment of a vasoactive neuropeptide (calcitonin gene related peptide) in the trigeminal sensory projection to the intracranial arteries. J Neurosci Off J Soc Neurosci 8:2468–2476. 10.1523/JNEUROSCI.08-07-02468.1988 10.1523/JNEUROSCI.08-07-02468.1988 PMC6569540 2470872

[81] McIlvried LA, Albers K, Gold MS (2010) Distribution of artemin and GFRalpha3 labeled nerve fibers in the dura mater of rat: artemin and GFRalpha3 in the dura. Headache 50:442–450. 10.1111/j.1526-4610.2009.01548.x 19845789 10.1111/j.1526-4610.2009.01548.x PMC3074600

[82] Keller JT, Marfurt CF (1991) Peptidergic and serotoninergic innervation of the rat dura mater. J Comp Neurol 309:515–534. 10.1002/cne.903090408 1717522 10.1002/cne.903090408

[83] Wang J, Xu D, Cui J et al (2021) Visualizing the Calcitonin Gene-Related Peptide Immunoreactive Innervation of the Rat Cranial Dura Mater with Immunofluorescence and Neural Tracing. J Vis Exp JoVE. 10.3791/61742 33491673 10.3791/61742

[84] Edvinsson L, Jansen I, Cunha e Sa M, Gulbenkian S (1994) Demonstration of neuropeptide containing nerves and vasomotor responses to perivascular peptides in human cerebral arteries. Cephalalgia Int J Headache 14:88–96. 10.1046/j.1468-2982.1994.1402088.x 10.1046/j.1468-2982.1994.1402088.x 7520366

[85] Wellman GC, Quayle JM, Standen NB (1998) ATP-sensitive K+ channel activation by calcitonin gene-related peptide and protein kinase A in pig coronary arterial smooth muscle. J Physiol 507(Pt 1):117–129. 10.1111/j.1469-7793.1998.117bu.x 9490826 10.1111/j.1469-7793.1998.117bu.x PMC2230768

[86] Hosaka K, Rayner SE, von der Weid P-Y et al (2006) Calcitonin gene-related peptide activates different signaling pathways in mesenteric lymphatics of guinea pigs. Am J Physiol Heart Circ Physiol 290:H813–822. 10.1152/ajpheart.00543.2005 16172164 10.1152/ajpheart.00543.2005

[87] Syed AU, Koide M, Brayden JE, Wellman GC (2019) Tonic regulation of middle meningeal artery diameter by ATP-sensitive potassium channels. J Cereb Blood Flow Metab Off J Int Soc Cereb Blood Flow Metab 39:670–679. 10.1177/0271678X17749392 10.1177/0271678X17749392 PMC6446425 29260608

[88] Nelson E, Rennels M (1970) Innervation of intracranial arteries. Brain J Neurol 93:475–490. 10.1093/brain/93.3.475 10.1093/brain/93.3.475 4097005

[89] Sato T, Sato S, Suzuki J (1980) Correlation with superior cervical sympathetic ganglion and sympathetic nerve innervation of intracranial artery-electron microscopical studies. Brain Res 188:33–41. 10.1016/0006-8993(80)90554-5 7370759 10.1016/0006-8993(80)90554-5

[90] Suzuki N, Hardebo JE (1991) Anatomical basis for a parasympathetic and sensory innervation of the intracranial segment of the internal carotid artery in man. Possible implication for vascular headache. J Neurol Sci 104:19–31. 10.1016/0022-510x(91)90211-o 1717660 10.1016/0022-510x(91)90211-o

[91] Edvinsson L, Gulbenkian S, Barroso CP et al (1998) Innervation of the human middle meningeal artery: immunohistochemistry, ultrastructure, and role of endothelium for vasomotility. Peptides 19:1213–1225. 10.1016/s0196-9781(98)00066-7 9786171 10.1016/s0196-9781(98)00066-7

[92] Larsson LI, Edvinsson L, Fahrenkrug J et al (1976) Immunohistochemical localization of a vasodilatory polypeptide (VIP) in cerebrovascular nerves. Brain Res 113:400–404. 10.1016/0006-8993(76)90951-3 953744 10.1016/0006-8993(76)90951-3

[93] Hanko JH, Törnebrandt K, Hardebo JE et al (1986) Neuropeptide Y induces and modulates vasoconstriction in intracranial and peripheral vessels of animals and man. J Auton Pharmacol 6:117–124. 10.1111/j.1474-8673.1986.tb00638.x 3755439 10.1111/j.1474-8673.1986.tb00638.x

[94] Suzuki N, Hardebo JE, Owman C (1988) Origins and pathways of cerebrovascular vasoactive intestinal polypeptide-positive nerves in rat. J Cereb Blood Flow Metab Off J Int Soc Cereb Blood Flow Metab 8:697–712. 10.1038/jcbfm.1988.117 10.1038/jcbfm.1988.117 3417797

[95] Fahrenkrug J, Hannibal J, Tams J, Georg B (2000) Immunohistochemical localization of the VIP1 receptor (VPAC1R) in rat cerebral blood vessels: relation to PACAP and VIP containing nerves. J Cereb Blood Flow Metab Off J Int Soc Cereb Blood Flow Metab 20:1205–1214. 10.1097/00004647-200008000-00006 10.1097/00004647-200008000-00006 10950381

[96] Lennerz JK, Rühle V, Ceppa EP et al (2008) Calcitonin receptor-like receptor (CLR), receptor activity-modifying protein 1 (RAMP1), and calcitonin gene-related peptide (CGRP) immunoreactivity in the rat trigeminovascular system: differences between peripheral and central CGRP receptor distribution. J Comp Neurol 507:1277–1299. 10.1002/cne.21607 18186028 10.1002/cne.21607

[97] Eftekhari S, Salvatore CA, Calamari A et al (2010) Differential distribution of calcitonin gene-related peptide and its receptor components in the human trigeminal ganglion. Neuroscience 169:683–696. 10.1016/j.neuroscience.2010.05.016 20472035 10.1016/j.neuroscience.2010.05.016

[98] Eftekhari S, Edvinsson L (2011) Calcitonin gene-related peptide (CGRP) and its receptor components in human and rat spinal trigeminal nucleus and spinal cord at C1-level. BMC Neurosci 12:112. 10.1186/1471-2202-12-112 22074408 10.1186/1471-2202-12-112 PMC3282678

[99] Maddahi A, Edvinsson JCA, Edvinsson L (2024) Sex differences in expression of CGRP family of receptors and ligands in the rat trigeminal system. J Headache Pain 25:193. 10.1186/s10194-024-01893-1 39516766 10.1186/s10194-024-01893-1 PMC11545840

[100] Edvinsson L, Ho TW (2010) CGRP receptor antagonism and migraine. Neurother J Am Soc Exp Neurother 7:164–175. 10.1016/j.nurt.2010.02.004 10.1016/j.nurt.2010.02.004 PMC5084097 20430315

[101] Zheng F, Nixdorf-Bergweiler BE, van Brederode J et al (2021) Excitatory Effects of Calcitonin Gene-Related Peptide (CGRP) on Superficial Sp5C Neurons in Mouse Medullary Slices. Int J Mol Sci 22:3794. 10.3390/ijms22073794 33917574 10.3390/ijms22073794 PMC8038766

[102] Hirata Y, Takagi Y, Takata S et al (1988) Calcitonin gene-related peptide receptor in cultured vascular smooth muscle and endothelial cells. Biochem Biophys Res Commun 151:1113–1121. 10.1016/s0006-291x(88)80481-9 2833256 10.1016/s0006-291x(88)80481-9

[103] Sams A, Knyihár-Csillik E, Engberg J et al (2000) CGRP and adrenomedullin receptor populations in human cerebral arteries: in vitro pharmacological and molecular investigations in different artery sizes. Eur J Pharmacol 408:183–193. 10.1016/s0014-2999(00)00781-0 11080525 10.1016/s0014-2999(00)00781-0

[104] Yang L, Xu M, Bhuiyan SA et al (2022) Human and mouse trigeminal ganglia cell atlas implicates multiple cell types in migraine. Neuron 110:1806–1821e8. 10.1016/j.neuron.2022.03.003 35349784 10.1016/j.neuron.2022.03.003 PMC9338779

[105] Bhuiyan SA, Xu M, Yang L et al (2024) Harmonized cross-species cell atlases of trigeminal and dorsal root ganglia. Sci Adv 10:eadj9173. 10.1126/sciadv.adj9173 38905344 10.1126/sciadv.adj9173 PMC11804847

[106] Nassini R, Materazzi S, Vriens J et al (2012) The headache tree via umbellulone and TRPA1 activates the trigeminovascular system. Brain J Neurol 135:376–390. 10.1093/brain/awr272 10.1093/brain/awr272 22036959

[107] Koivisto A, Chapman H, Jalava N et al (2014) TRPA1: a transducer and amplifier of pain and inflammation. Basic Clin Pharmacol Toxicol 114:50–55. 10.1111/bcpt.12138 24102997 10.1111/bcpt.12138

[108] Eberhardt M, Dux M, Namer B et al (2014) H2S and NO cooperatively regulate vascular tone by activating a neuroendocrine HNO-TRPA1-CGRP signalling pathway. Nat Commun 5:4381. 10.1038/ncomms5381 25023795 10.1038/ncomms5381 PMC4104458

[109] Krüger-Genge A, Blocki A, Franke R-P, Jung F (2019) Vascular Endothelial Cell Biology: An Update. Int J Mol Sci 20:4411. 10.3390/ijms20184411 31500313 10.3390/ijms20184411 PMC6769656

[110] Dux M, Will C, Vogler B et al (2015) Meningeal blood flow is controlled by H2 S-NO crosstalk activating HNO-TRPA1-CGRP signalling. Br J Pharmacol. 10.1111/bph.13164 25884403 10.1111/bph.13164 PMC4728414

[111] Stam AH, Haan J, van den Maagdenberg AMJM et al (2009) Migraine and genetic and acquired vasculopathies. Cephalalgia Int J Headache 29:1006–1017. 10.1111/j.1468-2982.2009.01940.x 10.1111/j.1468-2982.2009.01940.x 19689610

[112] Rajan R, Khurana D, Lal V (2015) Interictal cerebral and systemic endothelial dysfunction in patients with migraine: a case-control study. J Neurol Neurosurg Psychiatry 86:1253–1257. 10.1136/jnnp-2014-309571 25550413 10.1136/jnnp-2014-309571

[113] Domínguez-Vivero C, Leira Y, López-Ferreiro A et al (2020) Pentraxin 3 (PTX3): A Molecular Marker of Endothelial Dysfunction in Chronic Migraine. J Clin Med 9:849. 10.3390/jcm9030849 32244987 10.3390/jcm9030849 PMC7141491

[114] González-Quintanilla V, Toriello M, Palacio E et al (2016) Systemic and cerebral endothelial dysfunction in chronic migraine. A case-control study with an active comparator. Cephalalgia Int J Headache 36:552–560. 10.1177/0333102415607857 10.1177/0333102415607857 26395894

[115] Paolucci M, Altamura C, Vernieri F (2021) The Role of Endothelial Dysfunction in the Pathophysiology and Cerebrovascular Effects of Migraine: A Narrative Review. J Clin Neurol 17:164–175. 10.3988/jcn.2021.17.2.164 33835736 10.3988/jcn.2021.17.2.164 PMC8053543

[116] Gonçalves FM, Martins-Oliveira A, Speciali JG et al (2011) Endothelial nitric oxide synthase haplotypes associated with aura in patients with migraine. DNA Cell Biol 30:363–369. 10.1089/dna.2010.1152 21332392 10.1089/dna.2010.1152

[117] Eröz R, Bahadir A, Dikici S, Tasdemir S (2014) Association of endothelial nitric oxide synthase gene polymorphisms (894G/T, -786T/C, G10T) and clinical findings in patients with migraine. Neuromolecular Med 16:587–593. 10.1007/s12017-014-8311-0 24845269 10.1007/s12017-014-8311-0

[118] Dong H, Wang ZH, Dong B et al (2018) Endothelial nitric oxide synthase (-786T > C) polymorphism and migraine susceptibility: A meta-analysis. Med (Baltim) 97:e12241. 10.1097/MD.0000000000012241 10.1097/MD.0000000000012241 PMC6133471 30200152

[119] Borończyk M, Zduńska A, Węgrzynek-Gallina J et al (2025) Migraine and stroke: correlation, coexistence, dependence - a modern perspective. J Headache Pain 26:39. 10.1186/s10194-025-01973-w 39979846 10.1186/s10194-025-01973-w PMC11844069

[120] Lassen LH, Haderslev PA, Jacobsen VB et al (2002) CGRP may play a causative role in migraine. Cephalalgia Int J Headache 22:54–61. 10.1046/j.1468-2982.2002.00310.x 10.1046/j.1468-2982.2002.00310.x 11993614

[121] Edvinsson L, Goadsby PJ (1990) Extracerebral manifestations in migraine. A peptidergic involvement? J Intern Med 228:299–304. 10.1111/j.1365-2796.1990.tb00236.x 2266336 10.1111/j.1365-2796.1990.tb00236.x

[122] Sarchielli P, Alberti A, Codini M et al (2000) Nitric oxide metabolites, prostaglandins and trigeminal vasoactive peptides in internal jugular vein blood during spontaneous migraine attacks. Cephalalgia Int J Headache 20:907–918. 10.1046/j.1468-2982.2000.00146.x 10.1046/j.1468-2982.2000.00146.x 11304026

[123] Brain SD, Grant AD (2004) Vascular Actions of Calcitonin Gene-Related Peptide and Adrenomedullin. Physiol Rev 84:903–934. 10.1152/physrev.00037.2003 15269340 10.1152/physrev.00037.2003

[124] Nelson-Maney NP, Bálint L, Beeson ALS et al (2024) Meningeal lymphatic CGRP signaling governs pain via cerebrospinal fluid efflux and neuroinflammation in migraine models. J Clin Invest 134:e175616. 10.1172/JCI175616 38743922 10.1172/JCI175616 PMC11290972

[125] Li G, Cao Y, Tang X et al (2022) The meningeal lymphatic vessels and the glymphatic system: Potential therapeutic targets in neurological disorders. J Cereb Blood Flow Metab 42:1364–1382. 10.1177/0271678X221098145 35484910 10.1177/0271678X221098145 PMC9274866

[126] Rasmussen MK, Mestre H, Nedergaard M (2018) The glymphatic pathway in neurological disorders. Lancet Neurol 17:1016–1024. 10.1016/S1474-4422(18)30318-1 30353860 10.1016/S1474-4422(18)30318-1 PMC6261373

[127] Rasmussen MK, Mestre H, Nedergaard M (2022) Fluid transport in the brain. Physiol Rev 102:1025–1151. 10.1152/physrev.00031.2020 33949874 10.1152/physrev.00031.2020 PMC8897154

[128] Giniatullin R, Nistri A (2023) Role of ATP in migraine mechanisms: focus on P2X3 receptors. J Headache Pain 24:1. 10.1186/s10194-022-01535-4 36597043 10.1186/s10194-022-01535-4 PMC9809127

[129] Wang S, Chennupati R, Kaur H et al (2016) Endothelial cation channel PIEZO1 controls blood pressure by mediating flow-induced ATP release. J Clin Invest 126:4527–4536. 10.1172/JCI87343 10.1172/JCI87343 PMC5127677 27797339

[130] Park JI, Shin CY, Lee YW et al (2000) Endothelium-dependent sensory non-adrenergic non-cholinergic vasodilatation in rat thoracic aorta: involvement of ATP and a role for NO. J Pharm Pharmacol 52:409–416. 10.1211/0022357001774165 10813551 10.1211/0022357001774165

[131] Buvinic S, Briones R, Huidobro-Toro JP (2002) P2Y(1) and P2Y(2) receptors are coupled to the NO/cGMP pathway to vasodilate the rat arterial mesenteric bed. Br J Pharmacol 136:847–856. 10.1038/sj.bjp.0704789 12110609 10.1038/sj.bjp.0704789 PMC1573418

[132] Spehr J, Spehr M, Hatt H, Wetzel CH (2004) Subunit-specific P2X-receptor expression defines chemosensory properties of trigeminal neurons. Eur J Neurosci 19:2497–2510. 10.1111/j.0953-816X.2004.03329.x 15128403 10.1111/j.0953-816X.2004.03329.x

[133] Luo J, Yin G-F, Gu Y-Z et al (2006) Characterization of three types of ATP-activated current in relation to P2X subunits in rat trigeminal ganglion neurons. Brain Res 1115:9–15. 10.1016/j.brainres.2006.07.084 16934235 10.1016/j.brainres.2006.07.084

[134] Fabbretti E, D’Arco M, Fabbro A et al (2006) Delayed upregulation of ATP P2X3 receptors of trigeminal sensory neurons by calcitonin gene-related peptide. J Neurosci Off J Soc Neurosci 26:6163–6171. 10.1523/JNEUROSCI.0647-06.2006 10.1523/JNEUROSCI.0647-06.2006 PMC6675180 16763024

[135] Giniatullin R, Nistri A, Fabbretti E (2008) Molecular mechanisms of sensitization of pain-transducing P2X3 receptors by the migraine mediators CGRP and NGF. Mol Neurobiol 37:83–90. 10.1007/s12035-008-8020-5 18459072 10.1007/s12035-008-8020-5

[136] Simonetti M, Giniatullin R, Fabbretti E (2008) Mechanisms mediating the enhanced gene transcription of P2X3 receptor by calcitonin gene-related peptide in trigeminal sensory neurons. J Biol Chem 283:18743–18752. 10.1074/jbc.M800296200 18460469 10.1074/jbc.M800296200

[137] Schwenger N, Dux M, de Col R et al (2007) Interaction of calcitonin gene-related peptide, nitric oxide and histamine release in neurogenic blood flow and afferent activation in the rat cranial dura mater. Cephalalgia Int J Headache 27:481–491. 10.1111/j.1468-2982.2007.01321.x 10.1111/j.1468-2982.2007.01321.x 17441973

[138] Zhang X-C, Levy D (2008) Modulation of meningeal nociceptors mechanosensitivity by peripheral proteinase-activated receptor-2: the role of mast cells. Cephalalgia Int J Headache 28:276–284. 10.1111/j.1468-2982.2007.01523.x 10.1111/j.1468-2982.2007.01523.x PMC2504502 18254896

[139] Koroleva K, Gafurov O, Guselnikova V et al (2019) Meningeal Mast Cells Contribute to ATP-Induced Nociceptive Firing in Trigeminal Nerve Terminals: Direct and Indirect Purinergic Mechanisms Triggering Migraine Pain. Front Cell Neurosci 13:195. 10.3389/fncel.2019.00195 31133812 10.3389/fncel.2019.00195 PMC6524559

[140] Koroleva K, Ermakova E, Mustafina A et al (2020) Protective Effects of Hydrogen Sulfide Against the ATP-Induced Meningeal Nociception. Front Cell Neurosci 14:266. 10.3389/fncel.2020.00266 32982692 10.3389/fncel.2020.00266 PMC7492747

[141] Melo-Carrillo A, Strassman A, Burstein R (2026) Elucidating the nociceptive role of CGRP in migraine headache. Brain J Neurol. 10.1093/brain/awag008. awag008 10.1093/brain/awag008 PMC13431663 41503630

[142] Roch M, Messlinger K, Kulchitsky V et al (2007) Ongoing activity in trigeminal wide-dynamic range neurons is driven from the periphery. Neuroscience 150:681–691. 10.1016/j.neuroscience.2007.09.032 18023985 10.1016/j.neuroscience.2007.09.032

[143] Lambert GA, Truong L, Zagami AS (2011) Effect of cortical spreading depression on basal and evoked traffic in the trigeminovascular sensory system. Cephalalgia Int J Headache 31:1439–1451. 10.1177/0333102411422383 10.1177/0333102411422383 21940490

[144] Eftekhari S, Salvatore CA, Johansson S et al (2015) Localization of CGRP, CGRP receptor, PACAP and glutamate in trigeminal ganglion. Relation to the blood-brain barrier. Brain Res 1600:93–109. 10.1016/j.brainres.2014.11.031 25463029 10.1016/j.brainres.2014.11.031

[145] Edvinsson JCA, Warfvinge K, Krause DN et al (2019) C-fibers may modulate adjacent Aδ-fibers through axon-axon CGRP signaling at nodes of Ranvier in the trigeminal system. J Headache Pain 20:105. 10.1186/s10194-019-1055-3 31718551 10.1186/s10194-019-1055-3 PMC6852900

[146] Askwith T, Zeng W, Eggo MC, Stevens MJ (2012) Taurine reduces nitrosative stress and nitric oxide synthase expression in high glucose-exposed human Schwann cells. Exp Neurol 233:154–162. 10.1016/j.expneurol.2011.09.010 21952043 10.1016/j.expneurol.2011.09.010 PMC3268940

[147] Takahashi M, Kawaguchi M, Shimada K et al (2004) Cyclooxygenase-2 expression in Schwann cells and macrophages in the sciatic nerve after single spinal nerve injury in rats. Neurosci Lett 363:203–206. 10.1016/j.neulet.2004.03.040 15182944 10.1016/j.neulet.2004.03.040

[148] Berti-Mattera LN, Wilkins PL, Madhun Z, Suchovsky D (1996) P2-purigenic receptors regulate phospholipase C and adenylate cyclase activities in immortalized Schwann cells. Biochem J 314(Pt 2):555–561. 10.1042/bj3140555 8670070 10.1042/bj3140555 PMC1217085

[149] Kobayashi K, Fukuoka T, Yamanaka H et al (2006) Neurons and glial cells differentially express P2Y receptor mRNAs in the rat dorsal root ganglion and spinal cord. J Comp Neurol 498:443–454. 10.1002/cne.21066 16874807 10.1002/cne.21066

[150] Wang M, Duong TL, Rea BJ et al (2022) CGRP Administration Into the Cerebellum Evokes Light Aversion, Tactile Hypersensitivity, and Nociceptive Squint in Mice. Front Pain Res 3:861598. 10.3389/fpain.2022.861598 10.3389/fpain.2022.861598 PMC9082264 35547239

[151] Sowers LP, Wang M, Rea BJ et al (2020) Stimulation of Posterior Thalamic Nuclei Induces Photophobic Behavior in Mice. Headache J Head Face Pain 60:1961–1981. 10.1111/head.13917 10.1111/head.13917 PMC7604789 32750230

[152] Kuburas A, Russo AF (2023) Shared and independent roles of CGRP and PACAP in migraine pathophysiology. J Headache Pain 24:34. 10.1186/s10194-023-01569-2 37009867 10.1186/s10194-023-01569-2 PMC10069045

[153] De Logu F, Li Puma S, Landini L et al (2019) Schwann cells expressing nociceptive channel TRPA1 orchestrate ethanol-evoked neuropathic pain in mice. J Clin Invest 129:5424–5441. 10.1172/JCI128022 31487269 10.1172/JCI128022 PMC6877331

[154] Landini L, Souza Monteiro de Araujo D, Chieca M et al (2023) Acetaldehyde via CGRP receptor and TRPA1 in Schwann cells mediates ethanol-evoked periorbital mechanical allodynia in mice: relevance for migraine. J Biomed Sci 30:28. 10.1186/s12929-023-00922-6 37101198 10.1186/s12929-023-00922-6 PMC10131321

[155] Brum ES, Landini L, Souza Monteiro de Araújo D et al (2025) Characterisation of periorbital mechanical allodynia in the reserpine-induced fibromyalgia model in mice: The role of the Schwann cell TRPA1/NOX1 signalling pathway. Free Radic Biol Med 229:289–299. 10.1016/j.freeradbiomed.2025.01.040 39842732 10.1016/j.freeradbiomed.2025.01.040

[156] De Logu F, De Siena G, Landini L et al (2023) Non-neuronal TRPA1 encodes mechanical allodynia associated with neurogenic inflammation and partial nerve injury in rats. Br J Pharmacol 180:1232–1246. 10.1111/bph.16005 36494916 10.1111/bph.16005

[157] Iannone LF, Nassini R, Patacchini R et al (2023) Neuronal and non-neuronal TRPA1 as therapeutic targets for pain and headache relief. Temperature 10:50–66. 10.1080/23328940.2022.2075218 10.1080/23328940.2022.2075218 PMC10177743 37187829

[158] Ogawa N, Kurokawa T, Mori Y (2016) Sensing of redox status by TRP channels. Cell Calcium 60:115–122. 10.1016/j.ceca.2016.02.009 26969190 10.1016/j.ceca.2016.02.009

[159] Sakaguchi R, Mori Y (2020) Transient receptor potential (TRP) channels: Biosensors for redox environmental stimuli and cellular status. Free Radic Biol Med 146:36–44. 10.1016/j.freeradbiomed.2019.10.415 31682917 10.1016/j.freeradbiomed.2019.10.415

[160] Melo-Carrillo A, Strassman AM, Broide R et al (2024) Novel insight into atogepant mechanisms of action in migraine prevention. Brain J Neurol 147:2884–2896. 10.1093/brain/awae062 10.1093/brain/awae062 PMC11292906 38411458

[161] Melo-Carrillo A, Strassman AM, Nir R-R et al (2017) Fremanezumab-A Humanized Monoclonal Anti-CGRP Antibody-Inhibits Thinly Myelinated (Aδ) But Not Unmyelinated (C) Meningeal Nociceptors. J Neurosci Off J Soc Neurosci 37:10587–10596. 10.1523/JNEUROSCI.2211-17.2017 10.1523/JNEUROSCI.2211-17.2017 PMC5666582 28972120

[162] Zoga V, Kawano T, Liang M-Y et al (2010) KATP channel subunits in rat dorsal root ganglia: alterations by painful axotomy. Mol Pain 6:6. 10.1186/1744-8069-6-6 20102598 10.1186/1744-8069-6-6 PMC2825500

[163] Raffaelli B, Do TP, Chaudhry BA et al (2024) Activation of ATP-sensitive potassium channels triggers migraine attacks independent of calcitonin gene-related peptide receptors: a randomized placebo-controlled trial. Cephalalgia Int J Headache 44:3331024231222916. 10.1177/03331024231222916 10.1177/03331024231222916 38181724

[164] Zhuang ZA, Al-Karagholi MA-M, Ashina H et al (2025) Effect of sumatriptan on ATP-sensitive potassium channel opening in migraine: A randomised controlled trial. Cephalalgia Int J Headache 45:3331024251341464. 10.1177/03331024251341464 10.1177/03331024251341464 40624944

[165] Mei H-R, Lam M, Kulkarni SR et al (2025) Meningeal K ATP channels contribute to behavioral responses in preclinical migraine models. Pain 166:398–407. 10.1097/j.pain.0000000000003385 39661370 10.1097/j.pain.0000000000003385 PMC11723816

[166] Christensen RH, Strassman AM, Ashina M et al (2025) Opening of ATP-sensitive potassium channels activates meningeal nociceptors: Implications for the origin of migraine headache. Cephalalgia Int J Headache 45:3331024251359237. 10.1177/03331024251359237 10.1177/03331024251359237 40785517

[167] Kokoti L, Al-Karagholi MA-M, Zhuang ZA et al (2024) Non-vascular ATP-sensitive potassium channel activation does not trigger migraine attacks: A randomized clinical trial. Cephalalgia Int J Headache 44:3331024241248211. 10.1177/03331024241248211 10.1177/03331024241248211 38729773

[168] Suttinont C, Maeno K, Yano M et al (2024) Role of Piezo2 in Schwann Cell Volume Regulation and Its Impact on Neurotrophic Release Regulation. Cell Physiol Biochem Int J Exp Cell Physiol Biochem Pharmacol 58:292–310. 10.33594/000000713 10.33594/000000713 38973197

[169] De Logu F, Nassini R, Hegron A et al (2022) Schwann cell endosome CGRP signals elicit periorbital mechanical allodynia in mice. Nat Commun 13:646. 10.1038/s41467-022-28204-z 35115501 10.1038/s41467-022-28204-z PMC8813987

[170] Kobayashi K, Fukuoka T, Obata K et al (2005) Distinct expression of TRPM8, TRPA1, and TRPV1 mRNAs in rat primary afferent neurons with adelta/c-fibers and colocalization with trk receptors. J Comp Neurol 493:596–606. 10.1002/cne.20794 16304633 10.1002/cne.20794

[171] Houben T, Loonen IC, Baca SM et al (2017) Optogenetic induction of cortical spreading depression in anesthetized and freely behaving mice. J Cereb Blood Flow Metab Off J Int Soc Cereb Blood Flow Metab 37:1641–1655. 10.1177/0271678X16645113 10.1177/0271678X16645113 PMC5435281 27107026

[172] Vitale M, Tottene A, Zarin Zadeh M et al (2023) Mechanisms of initiation of cortical spreading depression. J Headache Pain 24:105. 10.1186/s10194-023-01643-9 37553625 10.1186/s10194-023-01643-9 PMC10408042

[173] Lauritzen M (2001) Cortical spreading depression in migraine. Cephalalgia Int J Headache 21:757–760. 10.1111/j.1468-2982.2001.00244.x 10.1111/j.1468-2982.2001.00244.x 11595007

[174] Brennan KC, Beltrán-Parrazal L, López-Valdés HE et al (2007) Distinct vascular conduction with cortical spreading depression. J Neurophysiol 97:4143–4151. 10.1152/jn.00028.2007 17329631 10.1152/jn.00028.2007

[175] Wahl M, Lauritzen M, Schilling L (1987) Change of cerebrovascular reactivity after cortical spreading depression in cats and rats. Brain Res 411:72–80. 10.1016/0006-8993(87)90682-2 3607427 10.1016/0006-8993(87)90682-2

[176] Zhao HT, Tuohy MC, Chow D et al (2021) Neurovascular dynamics of repeated cortical spreading depolarizations after acute brain injury. Cell Rep 37:109794. 10.1016/j.celrep.2021.109794 34610299 10.1016/j.celrep.2021.109794 PMC8590206

[177] Lauritzen M (1994) Pathophysiology of the migraine aura. The spreading depression theory. Brain J Neurol 117(Pt 1):199–210. 10.1093/brain/117.1.199 10.1093/brain/117.1.199 7908596

[178] Charles AC, Baca SM (2013) Cortical spreading depression and migraine. Nat Rev Neurol 9:637–644. 10.1038/nrneurol.2013.192 24042483 10.1038/nrneurol.2013.192

[179] Dodick DW (2018) A phase-by-phase review of migraine pathophysiology. Headache 58 Suppl 14–16. 10.1111/head.13300 10.1111/head.13300 29697154

[180] Borgdorff P (2018) Arguments against the role of cortical spreading depression in migraine. Neurol Res 40:173–181. 10.1080/01616412.2018.1428406 29350588 10.1080/01616412.2018.1428406

[181] McLeod GA, Josephson CB, Engbers JDT et al (2025) Mapping the migraine: Intracranial recording of cortical spreading depression in migraine with aura. Headache J Head Face Pain 65:658–665. 10.1111/head.14907 10.1111/head.14907 40055980

[182] Aboghazleh R, Alkahmous B, Turan B, Tuncer MC (2022) Spreading depolarization: A phenomenon in the brain. Arch Ital Biol 160:28–41. 10.12871/000398292022123 35913391 10.12871/000398292022123

[183] Lauritzen M, Olesen J (1984) Regional cerebral blood flow during migraine attacks by Xenon-133 inhalation and emission tomography. Brain J Neurol 107(Pt 2):447–461. 10.1093/brain/107.2.447 10.1093/brain/107.2.447 6609739

[184] Olesen J (2024) Cerebral blood flow and arterial responses in migraine: history and future perspectives. J Headache Pain 25:222. 10.1186/s10194-024-01903-2 39701954 10.1186/s10194-024-01903-2 PMC11656563

[185] Olesen J, Friberg L, Olsen TS et al (1990) Timing and topography of cerebral blood flow, aura, and headache during migraine attacks. Ann Neurol 28:791–798. 10.1002/ana.410280610 2285266 10.1002/ana.410280610

[186] Zhang X, Levy D, Noseda R et al (2010) Activation of meningeal nociceptors by cortical spreading depression: implications for migraine with aura. J Neurosci Off J Soc Neurosci 30:8807–8814. 10.1523/JNEUROSCI.0511-10.2010 10.1523/JNEUROSCI.0511-10.2010 PMC2907647 20592202

[187] Zhang X, Levy D, Kainz V et al (2011) Activation of central trigeminovascular neurons by cortical spreading depression. Ann Neurol 69:855–865. 10.1002/ana.22329 21416489 10.1002/ana.22329 PMC3174689

[188] Zhao J, Levy D (2016) Cortical spreading depression promotes persistent mechanical sensitization of intracranial meningeal afferents: implications for the intracranial mechanosensitivity of migraine. eNeuro 3:ENEURO.0287-16.2016. 10.1523/ENEURO.0287-16.2016. 10.1523/ENEURO.0287-16.2016 PMC5242377 28127585

[189] Ebersberger A, Schaible HG, Averbeck B, Richter F (2001) Is there a correlation between spreading depression, neurogenic inflammation, and nociception that might cause migraine headache? Ann Neurol 49:7–13 11198299

[190] Close LN, Eftekhari S, Wang M et al (2019) Cortical spreading depression as a site of origin for migraine: Role of CGRP. Cephalalgia Int J Headache 39:428–434. 10.1177/0333102418774299 10.1177/0333102418774299 PMC7007998 29695168

[191] Jiang L, Ma D, Grubb BD, Wang M (2019) ROS/TRPA1/CGRP signaling mediates cortical spreading depression. J Headache Pain 20:25. 10.1186/s10194-019-0978-z 30841847 10.1186/s10194-019-0978-z PMC6734415

[192] Jin X, Morais A, Sasaki Y et al (2025) Small-molecule CGRP antagonist atogepant does not affect cortical spreading depression susceptibility in rats. J Headache Pain 26:177. 10.1186/s10194-025-02127-8 40764901 10.1186/s10194-025-02127-8 PMC12326636

[193] Filiz A, Tepe N, Eftekhari S et al (2019) CGRP receptor antagonist MK-8825 attenuates cortical spreading depression induced pain behavior. Cephalalgia Int J Headache 39:354–365. 10.1177/0333102417735845 10.1177/0333102417735845 28971699

[194] Sukhotinsky I, Dilekoz E, Moskowitz MA, Ayata C (2008) Hypoxia and hypotension transform the blood flow response to cortical spreading depression from hyperemia into hypoperfusion in the rat. J Cereb Blood Flow Metab Off J Int Soc Cereb Blood Flow Metab 28:1369–1376. 10.1038/jcbfm.2008.35 10.1038/jcbfm.2008.35 18446167

[195] Jaitovich A, Jourd’heuil D (2017) A Brief Overview of Nitric Oxide and Reactive Oxygen Species Signaling in Hypoxia-Induced Pulmonary Hypertension. In: Wang Y-X (ed) Pulmonary Vasculature Redox Signaling in Health and Disease. Springer International Publishing, Cham, pp 71–81 10.1007/978-3-319-63245-2_6 PMC5863727 29047082

[196] Miller TR, Shivashankar R, Mossa-Basha M, Gandhi D (2015) Reversible Cerebral Vasoconstriction Syndrome, Part 1: Epidemiology, Pathogenesis, and Clinical Course. AJNR Am J Neuroradiol 36:1392–1399. 10.3174/ajnr.A4214 25593203 10.3174/ajnr.A4214 PMC7964694

[197] Chen S-P, Wang S-J (2022) Pathophysiology of reversible cerebral vasoconstriction syndrome. J Biomed Sci 29:72. 10.1186/s12929-022-00857-4 36127720 10.1186/s12929-022-00857-4 PMC9489486

[198] O’Connor TP, van der Kooy D (1986) Pattern of intracranial and extracranial projections of trigeminal ganglion cells. J Neurosci Off J Soc Neurosci 6:2200–2207. 10.1523/JNEUROSCI.06-08-02200.1986 10.1523/JNEUROSCI.06-08-02200.1986 PMC6568767 3489082

[199] Xiao Y, Richter JA, Hurley JH (2008) Release of glutamate and CGRP from trigeminal ganglion neurons: Role of calcium channels and 5-HT1 receptor signaling. Mol Pain 4:12. 10.1186/1744-8069-4-12 18416824 10.1186/1744-8069-4-12 PMC2359740

[200] Eberhardt M, Neeb L, Vogel E-M et al (2009) Glyceroltrinitrate facilitates stimulated CGRP release but not gene expression of CGRP or its receptor components in rat trigeminal ganglia. Neuropeptides 43:483–489. 10.1016/j.npep.2009.09.002 19864020 10.1016/j.npep.2009.09.002

[201] Vogler B, Kuhn A, Mackenzie KD et al (2023) The Anti-Calcitonin Gene-Related Peptide (Anti-CGRP) Antibody Fremanezumab Reduces Trigeminal Neurons Immunoreactive to CGRP and CGRP Receptor Components in Rats. Int J Mol Sci 24:13471. 10.3390/ijms241713471 37686275 10.3390/ijms241713471 PMC10487893

[202] Reducha PV, Nielsen LKS, Jensen MN et al (2026) TRPM3 activation causes CGRP release in trigeminal neurons: Implications for migraine mechanisms. Headache 66:672–687. 10.1111/head.15082 41133435 10.1111/head.15082 PMC12951702

[203] Li J, Vause CV, Durham PL (2008) Calcitonin gene-related peptide stimulation of nitric oxide synthesis and release from trigeminal ganglion glial cells. Brain Res 1196:22–32. 10.1016/j.brainres.2007.12.028 18221935 10.1016/j.brainres.2007.12.028 PMC2268710

[204] Seiler K, Nusser JI, Lennerz JK et al (2013) Changes in calcitonin gene-related peptide (CGRP) receptor component and nitric oxide receptor (sGC) immunoreactivity in rat trigeminal ganglion following glyceroltrinitrate pretreatment. J Headache Pain 14:74. 10.1186/1129-2377-14-74 24004534 10.1186/1129-2377-14-74 PMC3847895

[205] Edvinsson L, Grell A-S, Warfvinge K (2020) Expression of the CGRP Family of Neuropeptides and their Receptors in the Trigeminal Ganglion. J Mol Neurosci MN 70:930–944. 10.1007/s12031-020-01493-z 32086679 10.1007/s12031-020-01493-z PMC7253526

[206] Ten Tusscher MP, Klooster J, Vrensen GF (1989) Satellite cells as blood-ganglion cell barrier in autonomic ganglia. Brain Res 490:95–102. 10.1016/0006-8993(89)90434-4 2474362 10.1016/0006-8993(89)90434-4

[207] Goto T, Iwai H, Kuramoto E, Yamanaka A (2017) Neuropeptides and ATP signaling in the trigeminal ganglion. Jpn Dent Sci Rev 53:117–124. 10.1016/j.jdsr.2017.01.003 29201256 10.1016/j.jdsr.2017.01.003 PMC5703691

[208] Vause CV, Durham PL (2009) CGRP stimulation of iNOS and NO release from trigeminal ganglion glial cells involves mitogen-activated protein kinase pathways. J Neurochem 110:811–821. 10.1111/j.1471-4159.2009.06154.x 19457095 10.1111/j.1471-4159.2009.06154.x PMC2748229

[209] Dux M, Will C, Vogler B et al (2015) Meningeal blood flow is controlled by H2 S-NO crosstalk activating a HNO-TRPA1-CGRP signalling pathway. Br J Pharmacol. 10.1111/bph.13164 25884403 10.1111/bph.13164 PMC4728414

[210] Matsuka Y, Neubert JK, Maidment NT, Spigelman I (2001) Concurrent release of ATP and substance P within guinea pig trigeminal ganglia in vivo. Brain Res 915:248–255. 10.1016/s0006-8993(01)02888-8 11595216 10.1016/s0006-8993(01)02888-8

[211] Kaag Rasmussen M, Møllgård K, Bork PAR et al (2024) Trigeminal ganglion neurons are directly activated by influx of CSF solutes in a migraine model. Science 385:80–86. 10.1126/science.adl0544 38963846 10.1126/science.adl0544

[212] Biella G, Panara C, Pecile A, Sotgiu ML (1991) Facilitatory role of calcitonin gene-related peptide (CGRP) on excitation induced by substance P (SP) and noxious stimuli in rat spinal dorsal horn neurons. An iontophoretic study in vivo. Brain Res 559:352–356. 10.1016/0006-8993(91)90024-p 1724408 10.1016/0006-8993(91)90024-p

[213] Messlinger K, Fischer MJM, Lennerz JK (2011) Neuropeptide effects in the trigeminal system: pathophysiology and clinical relevance in migraine. Keio J Med 60:82–89 21979827 10.2302/kjm.60.82

[214] Yarwood RE, Imlach WL, Lieu T et al (2017) Endosomal signaling of the receptor for calcitonin gene-related peptide mediates pain transmission. Proc Natl Acad Sci U S A 114:12309–12314. 10.1073/pnas.1706656114 29087309 10.1073/pnas.1706656114 PMC5699040

[215] Takhshid MA, Owji AA, Panjehshahin MR (2007) In vitro effects of adrenomedullin and calcitonin gene related peptide on the release of serotonin and amino acids from rat dorsal spinal cord. Neurosci Lett 420:193–197. 10.1016/j.neulet.2007.03.061 17532569 10.1016/j.neulet.2007.03.061

[216] Löken LS, Braz JM, Etlin A et al (2021) Contribution of dorsal horn CGRP-expressing interneurons to mechanical sensitivity. eLife 10:e59751. 10.7554/eLife.59751 34061020 10.7554/eLife.59751 PMC8245130

[217] Eftekhari S, Edvinsson L (2010) Possible sites of action of the new calcitonin gene-related peptide receptor antagonists. Ther Adv Neurol Disord 3:369–378. 10.1177/1756285610388343 21179597 10.1177/1756285610388343 PMC3002638

[218] Storer RJ, Akerman S, Goadsby PJ (2004) Calcitonin gene-related peptide (CGRP) modulates nociceptive trigeminovascular transmission in the cat. Br J Pharmacol 142:1171–1181. 10.1038/sj.bjp.0705807 15237097 10.1038/sj.bjp.0705807 PMC1575174

[219] Fischer MJM, Koulchitsky S, Messlinger K (2005) The nonpeptide calcitonin gene-related peptide receptor antagonist BIBN4096BS lowers the activity of neurons with meningeal input in the rat spinal trigeminal nucleus. J Neurosci Off J Soc Neurosci 25:5877–5883. 10.1523/JNEUROSCI.0869-05.2005 10.1523/JNEUROSCI.0869-05.2005 PMC6724801 15976076

[220] Sixt M-L, Messlinger K, Fischer MJM (2009) Calcitonin gene-related peptide receptor antagonist olcegepant acts in the spinal trigeminal nucleus. Brain J Neurol 132:3134–3141. 10.1093/brain/awp168 10.1093/brain/awp168 19737844

[221] Covasala O, Stirn SL, Albrecht S et al (2012) Calcitonin gene-related peptide receptors in rat trigeminal ganglion do not control spinal trigeminal activity. J Neurophysiol 108:431–440. 10.1152/jn.00167.2011 22539824 10.1152/jn.00167.2011

[222] Pinho-Ribeiro FA, Deng L, Neel DV et al (2023) Bacteria hijack a meningeal neuroimmune axis to facilitate brain invasion. Nature 615:472–481. 10.1038/s41586-023-05753-x 36859544 10.1038/s41586-023-05753-x PMC10593113

[223] Russell FA, King R, Smillie S-J et al (2014) Calcitonin gene-related peptide: physiology and pathophysiology. Physiol Rev 94:1099–1142. 10.1152/physrev.00034.2013 25287861 10.1152/physrev.00034.2013 PMC4187032

[224] Balcziak LK, Russo AF (2022) Dural Immune Cells, CGRP, and Migraine. Front Neurol 13:874193. 10.3389/fneur.2022.874193 35432179 10.3389/fneur.2022.874193 PMC9009415

[225] Adi T, Gold MS (2026) Meningeal neuropeptide and neuroimmune interactions in the context of migraine. J Headache Pain. 10.1186/s10194-026-02291-5 41723373 10.1186/s10194-026-02291-5 PMC13032340

[226] Assas BM, Pennock JI, Miyan JA (2014) Calcitonin gene-related peptide is a key neurotransmitter in the neuro-immune axis. Front Neurosci 8. 10.3389/fnins.2014.00023 10.3389/fnins.2014.00023 PMC3924554 24592205

[227] Zhang X-C, Kainz V, Jakubowski M et al (2009) Localization of COX-1 and COX-2 in the intracranial dura mater of the rat. Neurosci Lett 452:33–36. 10.1016/j.neulet.2009.01.032 19444941 10.1016/j.neulet.2009.01.032 PMC2775514

[228] Ottosson A, Edvinsson L (1997) Release of histamine from dural mast cells by substance P and calcitonin gene-related peptide. Cephalalgia Int J Headache 17:166–174 10.1046/j.1468-2982.1997.1703166.x 9170339

[229] Akerman S, Williamson DJ, Kaube H, Goadsby PJ (2002) The role of histamine in dural vessel dilation. Brain Res 956:96–102 12426051 10.1016/s0006-8993(02)03485-6

[230] Friedrich N, Németh K, Tanner M et al (2024) Anti-CGRP antibody galcanezumab modifies the function of the trigeminovascular nocisensor complex in the rat. J Headache Pain 25:9. 10.1186/s10194-024-01717-2 38243174 10.1186/s10194-024-01717-2 PMC10799508

[231] Dimlich RV, Keller JT, Strauss TA, Fritts MJ (1991) Linear arrays of homogeneous mast cells in the dura mater of the rat. J Neurocytol 20:485–503 1869885 10.1007/BF01252276

[232] Dimitriadou V, Rouleau A, Trung Tuong MD et al (1997) Functional relationships between sensory nerve fibers and mast cells of dura mater in normal and inflammatory conditions. Neuroscience 77:829–839 9070755 10.1016/s0306-4522(96)00488-5

[233] Rosas EP, Paz ST, Costa RF et al (2022) Histomorphometry of mast cells in the convexity of human intracranial dura mater. J Anat 240:724–734. 10.1111/joa.13585 34816423 10.1111/joa.13585 PMC8930819

[234] Markowitz S, Saito K, Buzzi MG, Moskowitz MA (1989) The development of neurogenic plasma extravasation in the rat dura mater does not depend upon the degranulation of mast cells. Brain Res 477:157–165. 10.1016/0006-8993(89)91403-0 2702481 10.1016/0006-8993(89)91403-0

[235] Moskowitz MA (1993) Neurogenic inflammation in the pathophysiology and treatment of migraine. Neurology 43:S16–20 8389008

[236] Spekker E, Tanaka M, Szabó Á, Vécsei L (2021) Neurogenic Inflammation: The Participant in Migraine and Recent Advancements in Translational Research. Biomedicines 10:76. 10.3390/biomedicines10010076 35052756 10.3390/biomedicines10010076 PMC8773152

[237] Barbanti P, Egeo G, Aurilia C et al (2014) Drugs targeting nitric oxide synthase for migraine treatment. Expert Opin Investig Drugs 23:1141–1148. 10.1517/13543784.2014.918953 10.1517/13543784.2014.918953 24818644

[238] Pradhan AA, Bertels Z, Akerman S (2018) Targeted Nitric Oxide Synthase Inhibitors for Migraine. Neurotherapeutics 15:391–401. 10.1007/s13311-018-0614-7 29516436 10.1007/s13311-018-0614-7 PMC5935643

[239] Ernstsen C, Obelitz-Ryom K, Kristensen DMB et al (2024) Mechanisms of GTN-induced migraine: Role of NOS isoforms, sGC and peroxynitrite in a migraine relevant mouse model. Cephalalgia 44:03331024241277542. 10.1177/03331024241277542 10.1177/03331024241277542 39314067

