# Supplementary material for: A delayed endothelial–glial mechanism for CGRP-induced migraine
Source: J Headache Pain. 2026 Jul 16;27(1):184. doi: 10.1186/s10194-026-02455-3 (PMC13386771; doi:10.1186/s10194-026-02455-3)
Supplement: Supplementary file 1 — Supplementary Material 1 [file 10194_2026_2455_MOESM1_ESM.pdf]

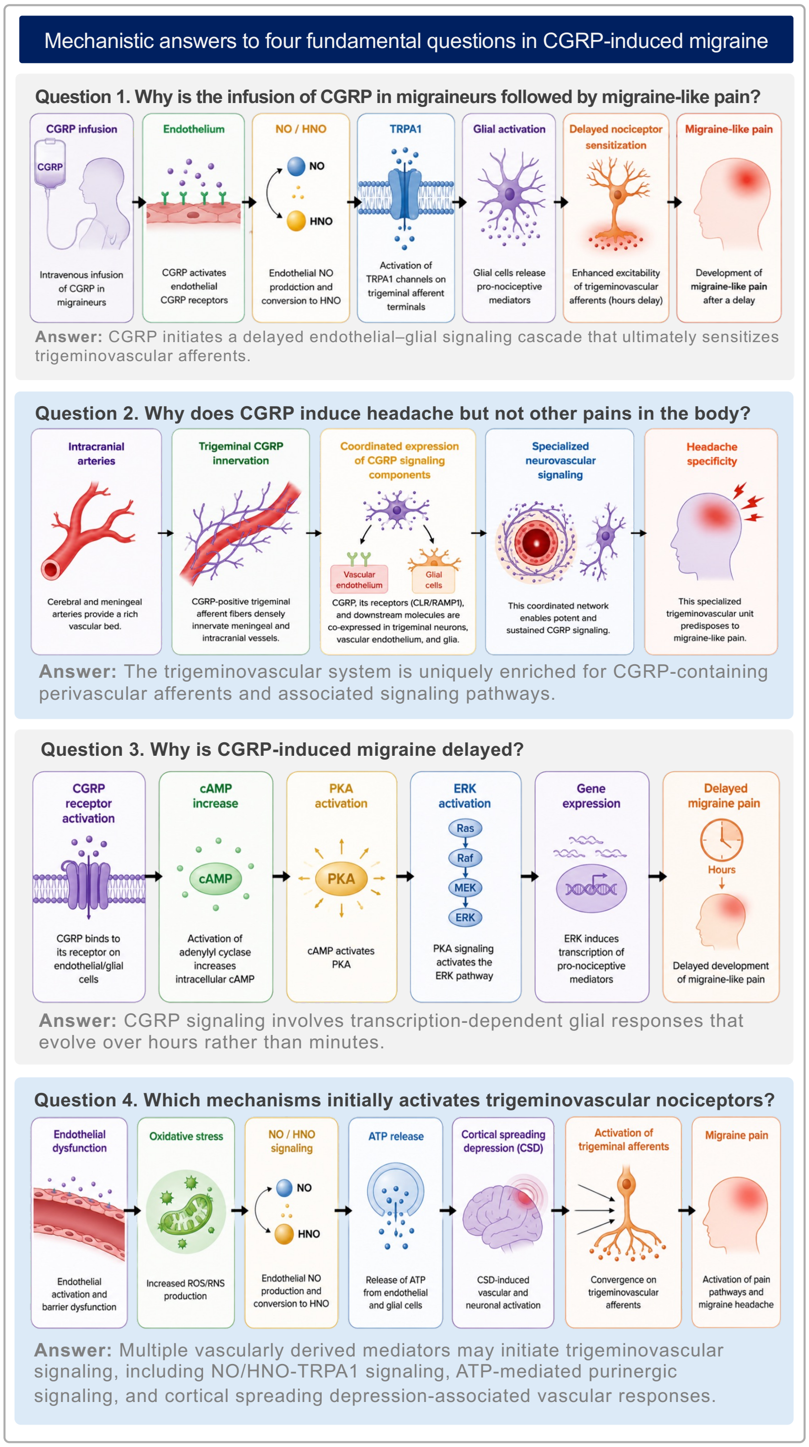
Mechanistic answers to four fundamental questions in CGRP-induced migraine
Question 1. Why is the infusion of CGRP in migraineurs followed by migraine-like pain?
CGRP infusion
CGRP
Intravenous infusion of CGRP in migraineurs
Endothelium
CGRP activates endothelial CGRP receptors
NO / HNO
NO
HNO
Endothelial NO prodoction and conversion to HNO
TRPA1
Activation of TRPA1 channels on trigeminal afferent terminals
Glial activation
Glial cells release pro-nociceptive mediators
Delayed nociceptor sensitization
Enhanced excitability of trigeminovascular afferents (hours delay)
Migraine-like pain
Development of migraine-like pain after a delay
Answer: CGRP initiates a delayed endothelial–glial signaling cascade that ultimately sensitizes trigeminovascular afferents.
Question 2. Why does CGRP induce headache but not other pains in the body?
Intracranial arteries
Cerebral and meningeal arteries provide a rich vascular bed.
Trigeminal CGRP innervation
CGRP-positive trigeminal afferent fibers densely innervate meningeal and intracranial vessels.
Coordinated expression of CGRP signaling components
Vascular endothelium
Glial cells
CGRP, its receptors (CLR/RAMP1), and downstream molecules are co-expressed in trigeminal neurons, vascular endothelium, and glia.
Specialized neurovascular signaling
This coordinated network enables potent and sustained CGRP signaling.
Headache specificity
This specialized trigeminovascular unit predisposes to migraine-like pain.
Answer: The trigeminovascular system is uniquely enriched for CGRP-containing perivascular afferents and associated signaling pathways.
Question 3. Why is CGRP-induced migraine delayed?
CGRP receptor activation
CGRP binds to its receptor on endothelial/glial cells
cAMP increase
cAMP
Activation of adenylyl cyclase increases intracellular cAMP
PKA activation
PKA
cAMP activates PKA
ERK activation
Ras
Raf
MEK
ERK
PKA signaling activates the ERK pathway
Gene expression
ERK induces transcription of pro-nociceptive mediators
Delayed migraine pain
Hours
Delayed development of migraine-like pain
Answer: CGRP signaling involves transcription-dependent glial responses that evolve over hours rather than minutes.
Question 4. Which mechanisms initially activates trigeminovascular nociceptors?
Endothelial dysfunction
Endothelial activation and barrier dysfunction
Oxidative stress
Increased ROS/RNS production
NO / HNO signaling
NO
HNO
Endothelial NO production and conversion to HNO
ATP release
Release of ATP from endothelial and glial cells
Cortical spreading depression (CSD)
CSD-induced vascular and neuronal activation
Activation of trigeminal afferents
Convergence on trigeminovascular afferents
Migraine pain
Activation of pain pathways and migraine headache
Answer: Multiple vascularly derived mediators may initiate trigeminovascular signaling, including NO/HNO-TRPA1 signaling, ATP-mediated purinergic signaling, and cortical spreading depression-associated vascular responses.
